# The Diversity of Aphidlion-like Larvae over the Last 130 Million Years

**DOI:** 10.3390/insects13040336

**Published:** 2022-03-30

**Authors:** Joachim T. Haug, Simon Linhart, Gideon T. Haug, Carsten Gröhn, Christel Hoffeins, Hans-Werner Hoffeins, Patrick Müller, Thomas Weiterschan, Jörg Wunderlich, Carolin Haug

**Affiliations:** 1 Faculty of Biology, Biocenter, Ludwig-Maximilians-Universität München (LMU Munich), Großhaderner Str. 2, 82152 Planegg-Martinsried, Germany; s.linhart@campus.lmu.de (S.L.); gideon.haug@palaeo-evo-devo.info (G.T.H.); chaug@bio.lmu.de (C.H.); 2 GeoBio-Center at LMU, Richard-Wagner-Str. 10, 80333 Munich, Germany; 3 Independent Researcher, 21509 Glinde, Germany; jcgroehn@t-online.de; 4 Independent Researcher, 22149 Hamburg, Germany; chw.hoffeins@googlemail.com (C.H.); hoffeins@aol.com (H.-W.H.); 5 Independent Researcher, 66482 Zweibrücken, Germany; pat14789@web.de; 6 Independent Researcher, 64739 Höchst im Odenwald, Germany; thomas.weiterschan@web.de; 7 Independent Researcher, 69493 Hirschberg, Germany; joergwunderlich@t-online.de

**Keywords:** Chrysopidae, Hemerobiidae, Neuroptera, Cretaceous, amber

## Abstract

**Simple Summary:**

The larvae of green lacewings and brown lacewings are called ‘aphidlions’, as they consume aphids. They play also an economic role as biological pest control. Aphidlions have, mostly, elongated spindle-shaped bodies, and similarly to most lacewing larvae, they possess a pair of venom-injecting compound jaws, also called stylets. Fossils that have been interpreted as aphidlions are known from amber of different ages (about 130, 100, 35, and 15 million years old). In this study, new aphidlion-like larvae are reported from about 100 million-year-old amber from Myanmar and about 35 million-year-old Baltic amber. The shapes of head and stylets were compared between the different time slices. With the newly described fossils and specimens from the literature, a total of 361 specimens could be included in the analysis: 78 fossil larvae, 188 extant larvae of brown lacewings, and 95 extant larvae of green lacewings. The results indicate that the diversity of head shapes stays about the same over time besides a certain increase in diversity of the head shapes in brown lacewing larvae. In certain other lacewings, a distinct decrease in the diversity of head shapes was observed in the larvae.

**Abstract:**

Aphidlions are larvae of certain lacewings (Neuroptera), and more precisely larvae of the groups Chrysopidae, green lacewings, and Hemerobiidae, brown lacewings. The name ‘aphidlion’ originates from their ecological function as specialised predators of aphids. Accordingly, they also play an economic role as biological pest control. Aphidlions have, mostly, elongated spindle-shaped bodies, and similarly to most lacewing larvae they are equipped with a pair of venom-injecting stylets. Fossils interpreted as aphidlions are known to be preserved in amber from the Cretaceous (130 and 100 million years ago), the Eocene (about 35 million years ago) and the Miocene (about 15 million years ago) ages. In this study, new aphidlion-like larvae are reported from Cretaceous amber from Myanmar (about 100 million years old) and Eocene Baltic amber. The shapes of head and stylets were compared between the different time slices. With the newly described fossils and specimens from the literature, a total of 361 specimens could be included in the analysis: 70 specimens from the Cretaceous, 5 from the Eocene, 3 from the Miocene, 188 extant larvae of Chrysopidae, and 95 extant larvae of Hemerobiidae. The results indicate that the diversity of head shapes remains largely unchanged over time, yet there is a certain increase in the diversity of head shapes in the larvae of Hemerobiidae. In certain other groups of Neuroptera, a distinct decrease in the diversity of head shapes in larval stages was observed.

## 1. Introduction

The group Holometabola, with well-known representatives, such as beetles, bees, butterflies, or flies, represents a large share of animal biodiversity, especially in terrestrial ecosystems. Moreover, some smaller lineages are part of this diversity, such as Neuroptera, the group of lacewings. Neuroptera is generally understood as having been part of the early radiation of Holometabola, and as more diverse and species-rich in the past [1,2,3]. For example, the now extinct group Kalligrammatidae seems to have played an important role in pollination back in the Mesozoic [4,5,6].

Many holometabolans spend most of their lifetime in their larval stages; this is also true for many lacewings. Hence, a supposed loss of biodiversity within a holometabolan ingroup should also be expected to manifest in a loss of the morphological and ecological diversity of larvae. Indeed, the Cretaceous has provided numerous lacewing larvae with peculiar morphologies, clearly not represented in the extant fauna [7,8,9,10,11,12,13,14,15,16,17,18,19,20] and indicating a loss of larval diversity after the Cretaceous.

Furthermore, a loss of diversity was demonstrated with quantitative measures of morphology for the larvae of silky lacewings (Psychopsidae, long-nosed antlions; [21]) and of split-footed lacewings (Nymphidae; [22]). In other lineages, quantitative comparisons yielded a less clear picture, as for spoon-winged lacewings (Nemopterinae; [23]), thread-winged lacewings (Crocinae; [24]) as well as different lineages of larvae with straight mouthparts [25].

A type of lacewing larva that is quite common in Cretaceous amber is that of the aphidlion. The term is used for extant larvae of the two groups Chrysopidae (green lacewings) and Hemerobiidae (brown lacewings). The name ‘aphidlion’ refers to the habit of larvae to consume aphids [26,27]. Hence the larvae occur on plants on which aphids also occur [28]. Representatives of Chrysopidae occur more or less worldwide apart from New Zealand; representatives of Hemerobiidae occur in temperate and tropical climates, yet they seem to have higher abundances in temperate regions [29].

Older studies still indicated a sister-group relationship between Chrysopidae and Hemerobiidae, mostly based on morphological characters (e.g., [30,31]), but also on molecular data [32]. Yet, the most recent studies resolved no closer relationship between the two groups [33,34,35,36]. Hence, it seems that the term aphidlion refers to a larval type with a specific ecological function and, coupled to this, with a certain morphology. Their ecological role makes the larvae interesting as ecological pest control [37].

The mouthparts of aphidlions differ from those of the more commonly known lacewing larvae, such as those of antlions, in being simpler. Similarly to other lacewings, each upper jaw (mandible) and lower jaw (maxilla) form a stylet [38]. These stylets are curved and inject venom and saliva into the prey and allow the sucking of the liquified tissues of the prey [38,39,40]. The stylets of antlion larvae bear distinct teeth; aphidlions lack such structures [18,41,42,43,44].

Many larvae of Chrysopidae camouflage themselves by attaching different objects to their back. This camouflaging cloak or package can be composed of rather different objects, including the remains of consumed prey, snail shells [45], or plant trichomes [46]. The camouflage can be used to deceive possible predators, but can also help to approach prey without being recognised. Larvae of *Chrysopa slossonae* can sneak across ants guarding their prey, as they use the wax of their pre as part of the camouflaging cloak [47]. 

Fossils of aphidlions with a very modern appearance are known from ambers from the Miocene [48,49,50] and the Eocene, with a clear record for Hemerobiidae [51,52] and a possible record for Chrysopidae [53], yet the latter seems to be partly unclear [54]. From the Cretaceous, numerous larvae have been interpreted to be closely related to Chrysopidae; such larvae clearly have an overall aphidlion-like morphology [7,8,9,10,11,12,13,20,55,56].

We here summarise all known occurrences of aphidlions and aphidlion-like lacewing larvae in the fossil record and report numerous new specimens from different ambers. Similar to earlier studies, we compare the morphological diversity of the heads of these larvae over time in order to recognise possible decreases of diversity.

## 2. Materials and Methods

### 2.1. Materials

Data for the analysis originated from different sources. Numerous specimens were based on images from the literature or databases (image repositories). Other specimens were directly inspected and documented. Extant specimens of aphidlions came from two zoological collections: the Zoologische Staatsammlung München (ZSM) and the Centrum für Naturkunde Hamburg (CeNak), Leibniz-Institut zur Analyse des Biodiversitätswandels (LIB). Fossil specimens, all preserved in amber, came from various collections: the Palaeo-Evo-Devo Research Group Collection of Arthropods, Ludwig-Maximilians-Universität München (PED), the Senckenberg Forschungsinstitut und Naturmuseum, Frankfurt/Main (SMF Be), and collections of some of the authors, namely C.G. (CCGG), Ch.H. + H.-W.H. (CCHH), P.M. (BUB), T.W. (Weiterschan BuB), and J.W. (F xxx BU CJW) (for details, see Files S1 and S2).

In total, 361 specimens were included into a shape analysis: 188 extant larvae of Chrysopidae, 95 extant larvae of Hemerobiidae, 3 larvae from the Miocene, 2 larvae of Chrysopidae from the Eocene, 3 larvae of Hemerobiidae from the Eocene, and 70 larvae from the Cretaceous. Details of the specimens are provided in File S1; for additional references, see File S2.

### 2.2. Documentation Methods

All fossil specimens were documented on a Keyence VHX-6000 digital microscope. Extant specimens were either documented on the same microscope or with a super-macro-photography set up.

Fossil specimens recorded on the VHX microscope were documented with different illumination settings, low-angle ring light and cross-polarised co-axial light, and in front of a white and a black background [17]. Images with the highest degree of detail were used for further analysis. 

The super-macro-photography set up included a Canon EOS 650D camera with an MP-E 65 mm macrolens. Illumination was either provided by macro-twin flashes or a pair of two single flashes (Yongnuo Digital Speedlite YN560EX II). Polarisers were placed on flashes and in front of the lens to provide cross-polarised light [57,58].

All images are composite images [59]; a stack of several frames of shifting focal planes was recorded for each image detail (processed with CombineZP or built-in software of microscope); adjacent images details were recorded (each with a stack) and merged to a large panorama (processed with Adobe Photoshop CS3, Adobe Photoshop Elements 11, or built-in software of microscope). Images recorded on VHX microscope are HDR images, additionally. The further processing of images (contrast, levels, and sharpness) was performed in Adobe Photoshop CS2.

### 2.3. Drawings

Outlines of all head capsules and stylets were redrawn in Adobe Illustrator CS2 or Inkscape. The more accessible half of the head capsule and the more accessible stylet were outlined. The stylet was artificially rotated forward so that the tip was in a straight line with the proximal insertion on the head capsule. Afterwards, the drawn half of the head was mirrored.

In cases in which the head was partly concealed by the thorax (the head was retracted) we only considered the visible or free part. This area reflects the functional part of the head, but also considers that for cases of fossils only the outer visible part can be considered. For many (but not all) extant specimens, often the entire head is additionally available, either in the literature or on cleared mounted specimens. Yet, to have an even dataset we did not consider this type of information, as it was not available for some of the other specimens.

### 2.4. Shape Analysis

The analysis was performed in SHAPE. The procedure was outlined in [60]. The application to lacewing larvae has been demonstrated in various recent studies [19,21,22,23,24,25].

## 3. Results

### 3.1. Short Descriptions of New Fossil Larvae

**Figure 1:**
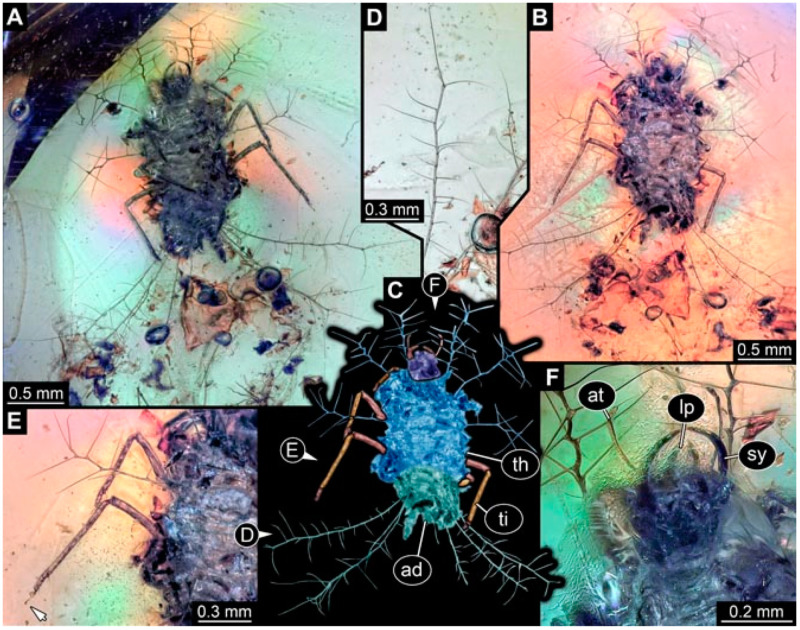
Specimen 4819 (BUB 3060); Myanmar amber. (**A**) Ventral view. (**B**) Dorsal view. (**C**) Dorsal view, colour-marked. (**D**) Close-up of protrusion in ventral view. (**E**) Close-up of trunk appendages with empodia (arrow). (**F**) Close-up of head capsule in ventral view. Abbreviations: ad = abdomen; at = antenna; lp = labial palp; sy = stylet; th = thorax; ti = tibia.

**Figure 2:**
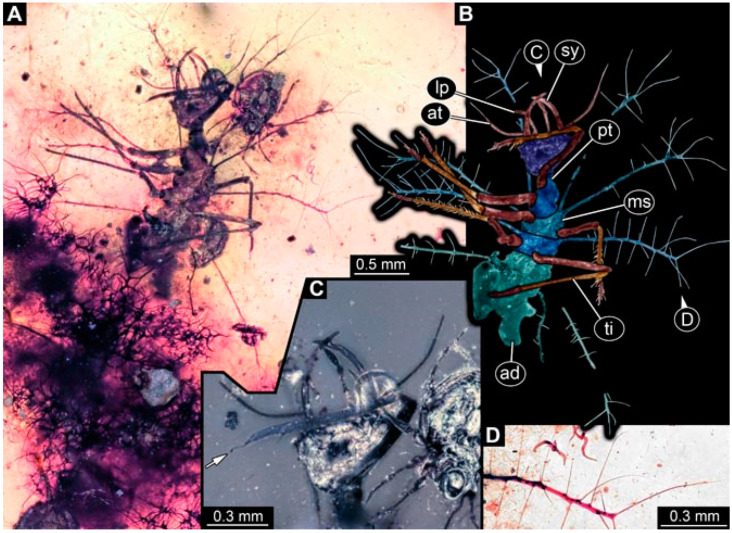
Specimen 4821 (BUB 3066); Myanmar amber. (**A**) Ventral view. (**B**) Ventral view, colour-marked. (**C**) Close-up of head capsule in ventral view and close-up of trunk appendage with empodium (arrow). (**D**) Close-up of protrusion in ventral view. Abbreviations: ad = abdomen; at = antenna; lp = labial palp; ms = mesothorax; pt = prothorax; sy = stylet; ti = tibia.

**Figure 3:**
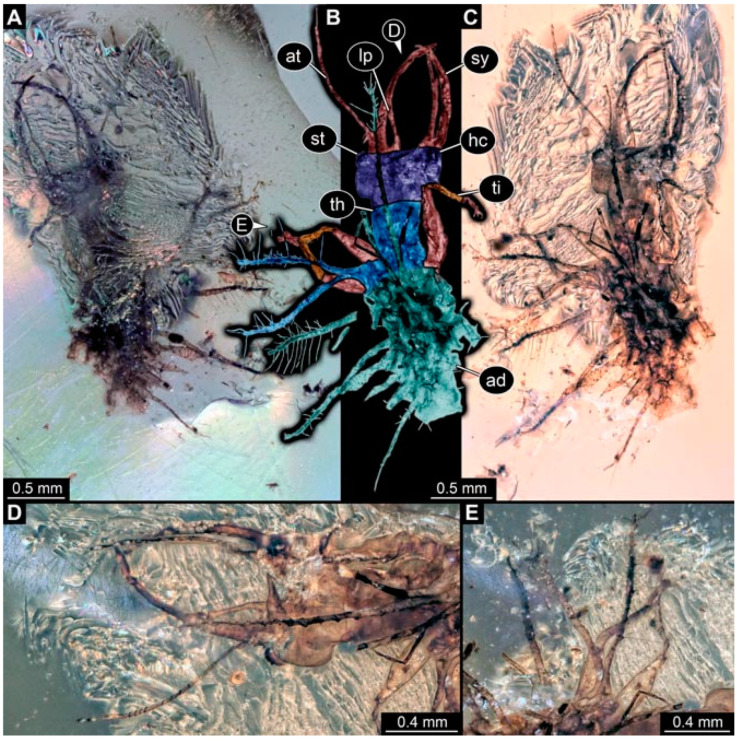
Specimen 4822 (BUB 3347); Myanmar amber. (**A**) Dorsal view. (**B**) Ventral view, colour-marked. (**C**) Ventral view. (**D**) Close-up of head capsule in ventral view. (**E**) Close-up of trunk appendages. Abbreviations: ad = abdomen; at = antenna; hc = head capsule; lp = labial palp; st = stemmata; sy = stylet; th = thorax; ti = tibia.

**Figure 4:**
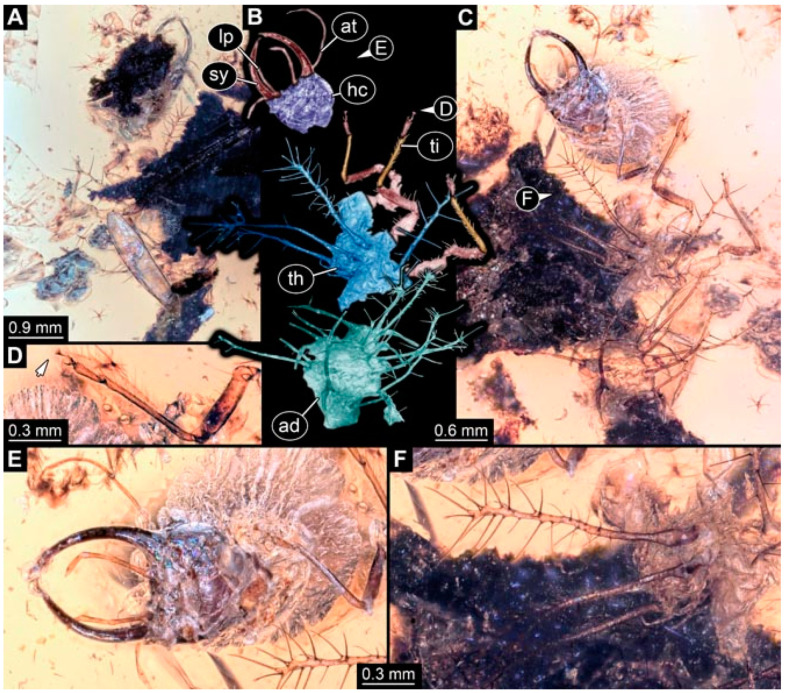
Specimen 4823 (BUB 3358); Myanmar amber. (**A**) Dorsal view. (**B**) Ventral view, colour-marked. (**C**) Ventral view. (**D**) Close-up of trunk appendage with empodium (arrow). (**E**) Close-up of head capsule in ventral view. (**F**) Close-up of protrusion in ventral view. Abbreviations: ad = abdomen; at = antenna; hc = head capsule; lp = labial palp; sy = stylet; th = thorax; ti = tibia.

**Figure 5:**
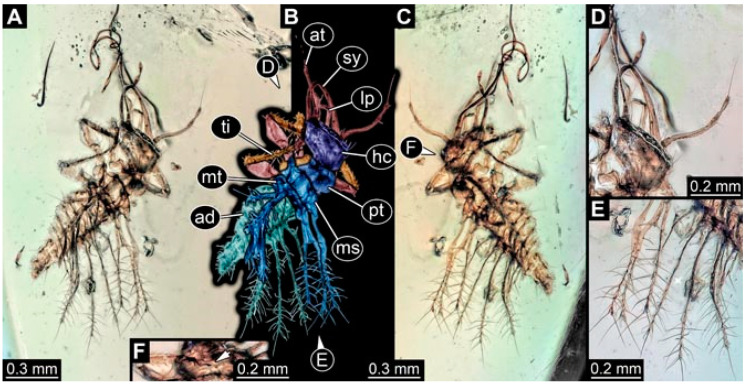
Specimen 4824 (BUB 3359); Myanmar amber. (**A**) Dorsal view. (**B**) Dorsal view, colour-marked. (**C**) Ventral view. (**D**) Close-up of head capsule in dorsal view. (**E**) Close-up of protrusions in ventral view. (**F**) Close-up of trunk appendage with empodium (arrows). Abbreviations: ad = abdomen; at = antenna; hc = head capsule; lp = labial palp; ms = mesothorax; mt = metathorax; pt = prothorax; sy = stylet; ti = tibia.

**Figure 6:**
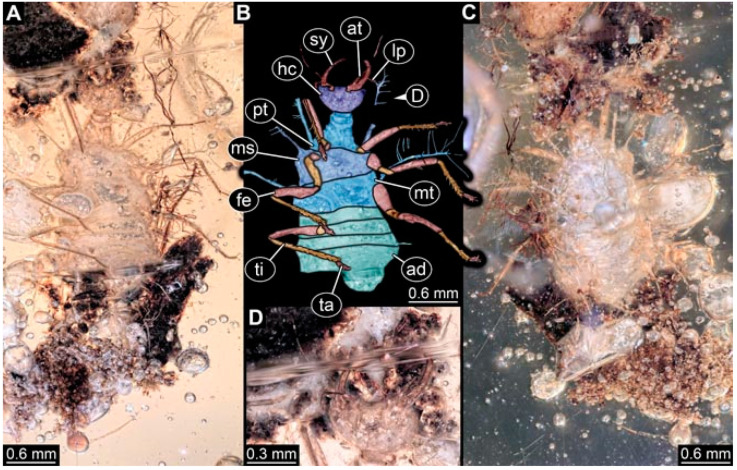
Specimen 4820 (BUB 3361); Myanmar amber. (**A**) Ventral view. (**B**) Ventral view, colour-marked. (**C**) Dorsal view. (**D**) Close-up of head capsule in ventral view. Abbreviations: ad = abdomen; at = antenna; fe = femur; hc = head capsule; lp = labial palp; ms = mesothorax; mt = metathorax; pt = prothorax; sy = stylet; ta = tarsus; ti = tibia.

**Figure 7:**
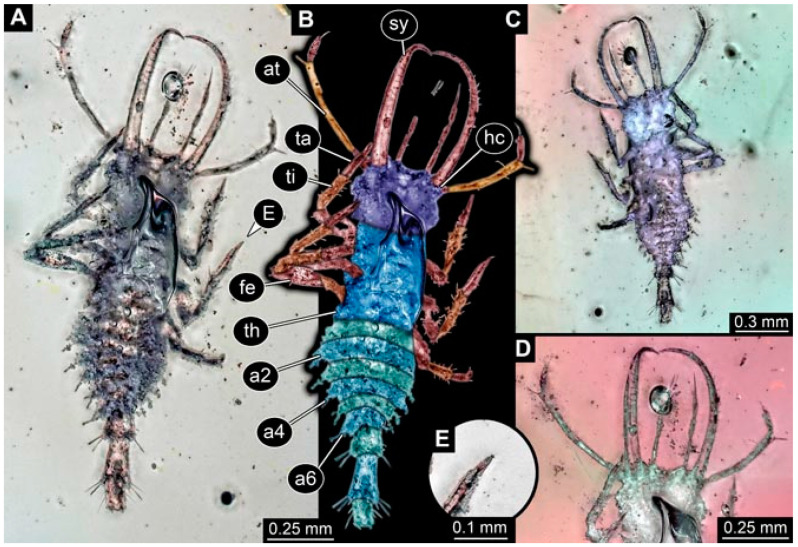
Specimen 4825 (BUB 3379); Myanmar amber. (**A**) Ventral view. (**B**) Ventral view, colour-marked. (**C**) Dorsal view. (**D**) Close-up of head capsule in ventral view. (**E**) Close-up of distal end of trunk appendage. Abbreviations: a2–a6 = abdomen segments 2–6; at = antenna; fe = femur; hc = head capsule; sy = stylet; ta = tarsus; th = thorax; ti = tibia.

**Figure 8:**
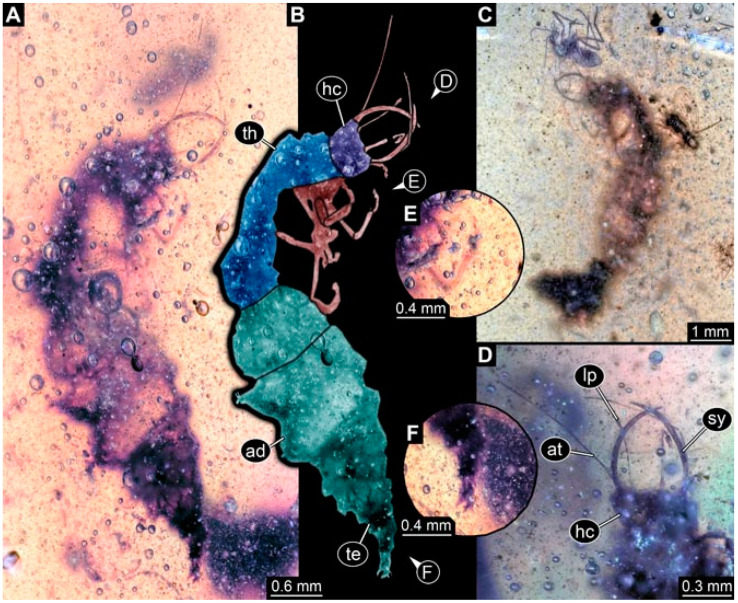
Specimen 4826 (BUB 3393); Myanmar amber. (**A**) Dorsal view. (**B**) Dorsal view, colour-marked. (**C**) Ventral view. (**D**) Close-up of head capsule in dorsal view. (**E**) Close-up of trunk appendage. (**F**) Close-up of trunk end in dorsal view. Abbreviations: ad = abdomen; at = antenna; hc = head capsule; lp = labial palp; sy = stylet; te = trunk end; th = thorax.

**Figure 9:**
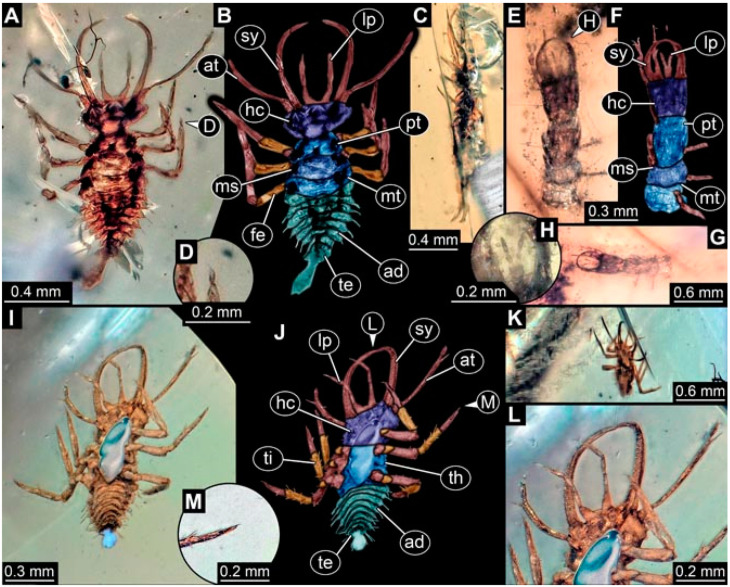
Three specimens in Myanmar amber. (**A**–**D**) Specimen 4827 (F 3196 BU CJW). (**A**) Dorsal view. (**B**) Dorsal view, colour-marked. (**C**) Lateral view. (**D**) Close-up of distal end of trunk appendage. (**E**–**H**) Specimen 4829 (PED 0038). (**E**) Dorsal view. (**F**) Dorsal view, colour-marked. (**G**) Ventral view. (**H**) Close-up of labium in dorsal view. (**I**–**M**) Specimen 4864 (PED 1223). (**I**) Ventral view. (**J**) Ventral view, colour-marked. (**K**) Dorsal view. (**L**) Close-up of head capsule in ventral view. (**M**) Close-up of distal end of trunk appendage. Abbreviations: ad = abdomen; at = antenna; fe = femur; hc = head capsule; lp = labial palp; ms = mesothorax; mt = metathorax; pt = prothorax; sy = stylet; te = trunk end; th = thorax; ti = tibia.

**Figure 10:**
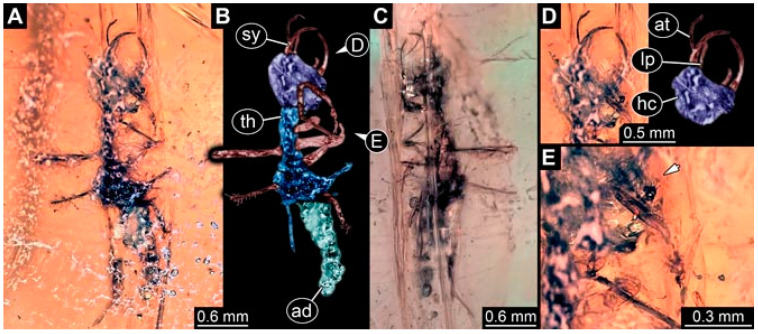
Specimen 4828 (PED 0034); Myanmar amber. (**A**) Ventral view. (**B**) Ventral view, colour-marked. (**C**) Dorsal view. (**D**) Close-up of head capsule in ventral view in original (**left**) and colour-marked version (**right**). (**E**) Close-up of trunk appendage with empodium (arrow). Abbreviations: ad = abdomen; at = antenna; hc = head capsule; lp = labial palp; sy = stylet; th = thorax.

**Figure 11:**
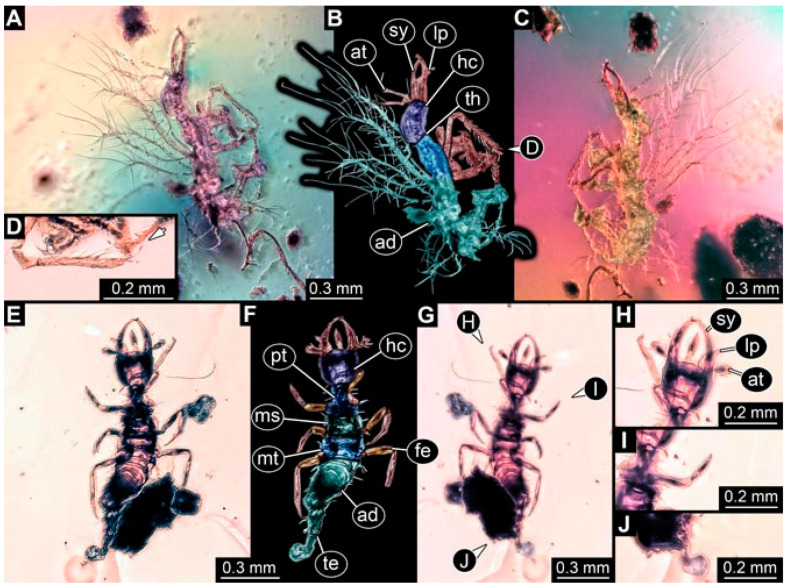
Two specimens in Myanmar amber. (**A**–**D**) Specimen 4830 (PED 0065). (**A**) Dorso-lateral view. (**B**) Dorso-lateral view, colour-marked. (**C**) Ventro-lateral view. (**D**) Close-up of trunk appendage with empodium (arrow). (**E**–**J**) Specimen 4832 (PED 0248). (**E**) Dorsal view. (**F**) Dorsal view, colour-marked. (**G**) Ventral view. (**H**) Close-up of head capsule in ventral view. (**I**) Close-up of trunk appendage. (**J**) Close-up of trunk end in ventral view. Abbreviations: ad = abdomen; at = antenna; fe = femur; hc = head capsule; lp = labial palp; ms = mesothorax; mt = metathorax; pt = prothorax; sy = stylet; te = trunk end; th = thorax.

**Figure 12:**
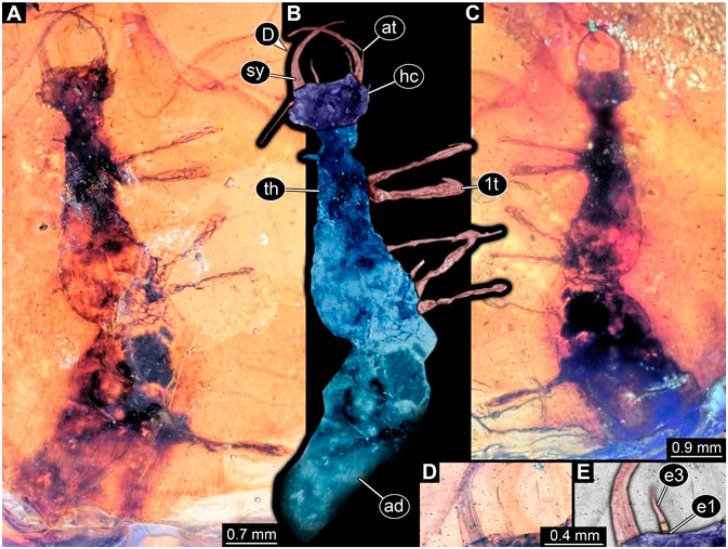
Specimen 4831 (PED 0149); Myanmar amber. (**A**) Dorsal view. (**B**) Dorsal view, colour-marked. (**C**) Ventral view. (**D**) Close-up of labial palp in dorsal view. (**E**) Close-up of labial palp in dorsal view, colour-marked. Abbreviations: 1t = first trunk appendage (walking leg); ad = abdomen; at = antenna; e1, e3 = element 1, 3; hc = head capsule; sy = stylet; th = thorax.

**Figure 13:**
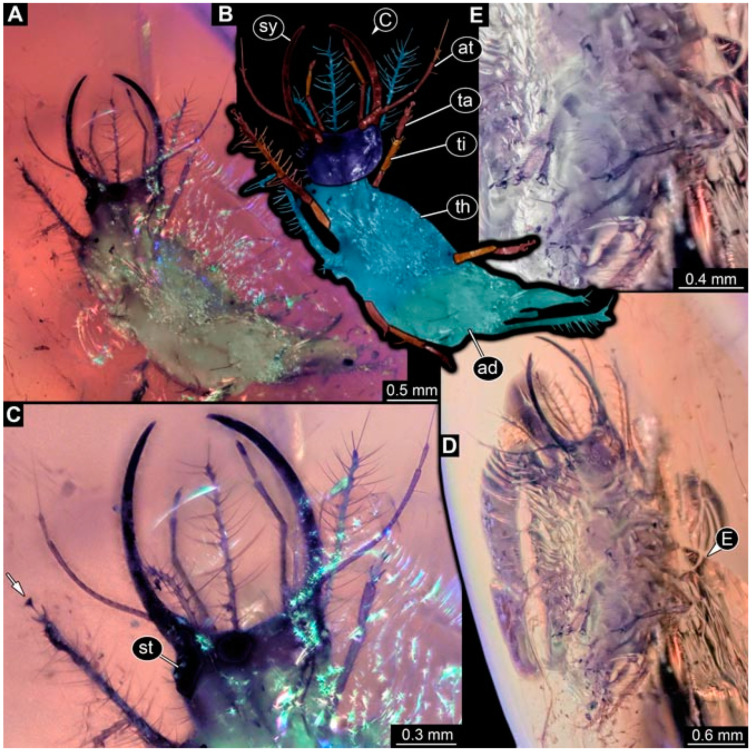
Specimen 4833 (PED 0251); Myanmar amber. (**A**) Dorsal view. (**B**) Dorsal view, colour-marked. (**C**) Close-up of head capsule in dorsal view and close-up of trunk appendage with empodium (arrow). (**D**) Ventral view. (**E**) Close-up of trunk appendages. Abbreviations: ad = abdomen; at = antenna; st = stemmata; sy = stylet; ta = tarsus; th = thorax; ti = tibia.

**Figure 14:**
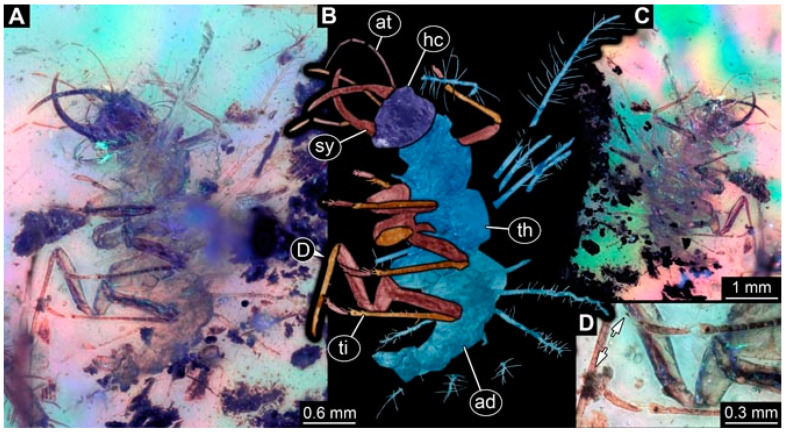
Specimen 4834 (PED 0252); Myanmar amber. (**A**) Dorso-lateral view. (**B**) Dorso-lateral view, colour-marked. (**C**) Ventral view. (**D**) Close-up of trunk appendages with empodia (arrows). Abbreviations: ad = abdomen; at = antenna; hc = head capsule; sy = stylet; th = thorax; ti = tibia.

**Figure 15:**
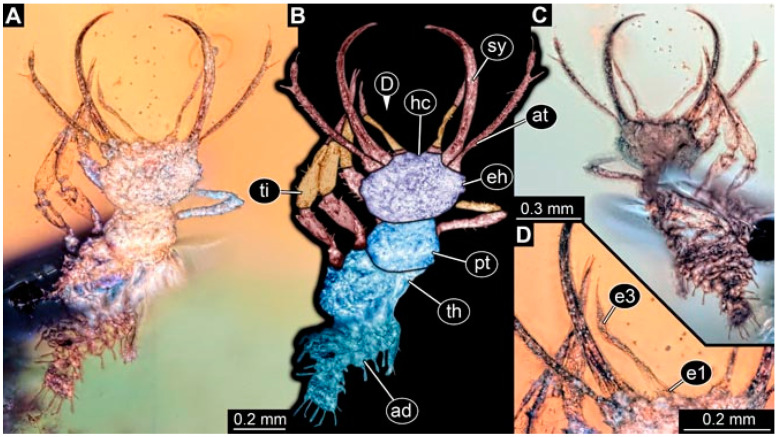
Specimen 4835 (PED 0253); Myanmar amber. (**A**) Dorsal view. (**B**) Dorsal view, colour-marked. (**C**) Ventral view. (**D**) Close-up of labial palp in dorsal view. Abbreviations: ad = abdomen; at = antenna; e1, e3 = element 1, 3; eh = eye hill; hc = head capsule; pt = prothorax; sy = stylet; th = thorax; ti = tibia.

**Figure 16:**
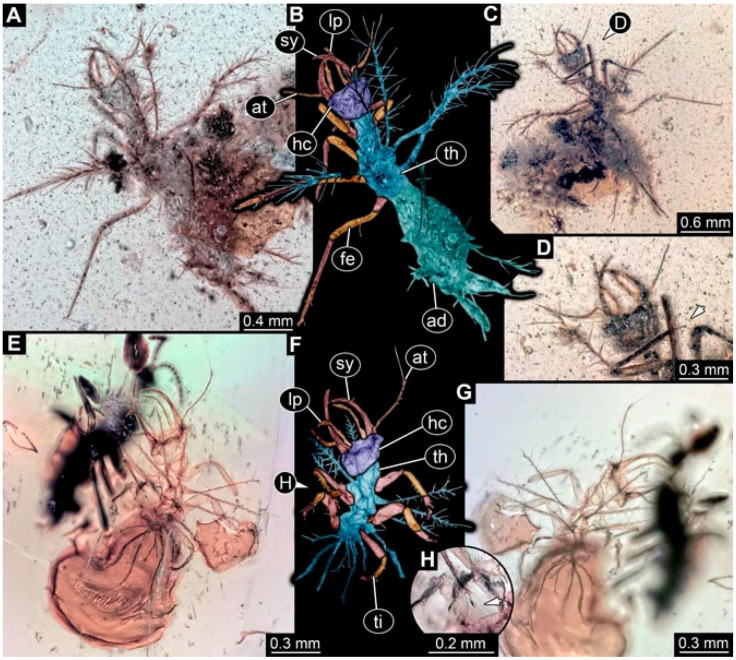
Two specimens in Myanmar amber. (**A**–**D**) Specimen 4836 (PED 0315). (**A**) Dorsal view. (**B**) Dorsal view, colour-marked. (**C**) Ventral view. (**D**) Close-up of trunk appendage with empodium (arrow). (**E**–**H**) Specimen 4837 (PED 0323). (**E**) Dorsal view. (**F**) Dorsal view, colour-marked. (**G**) Ventral view. (**H**) Close-up of trunk appendages with empodium (arrow). Abbreviations: ad = abdomen; at = antenna; fe = femur; hc = head capsule; lp = labial palp; sy = stylet; th = thorax; ti = tibia.

**Figure 17:**
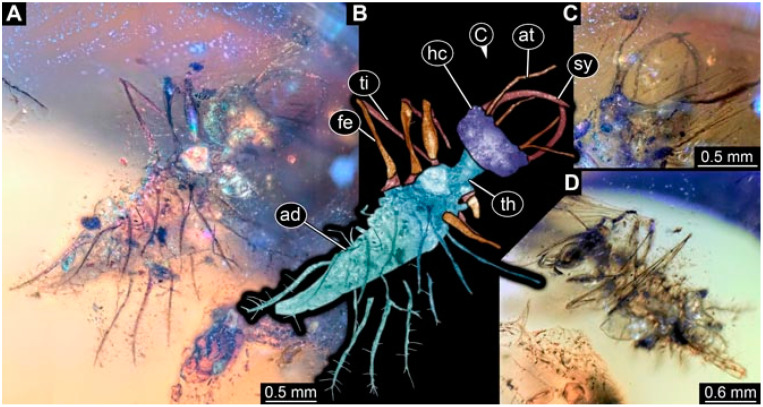
Specimen 4838 (PED 0330); Myanmar amber. (**A**) Dorsal view. (**B**) Dorsal view, colour-marked. (**C**) Close-up of head capsule in dorsal view. (**D**) Ventral view. Abbreviations: ad = abdomen; at = antenna; fe = femur; hc = head capsule; sy = stylet; th = thorax; ti = tibia.

**Figure 18:**
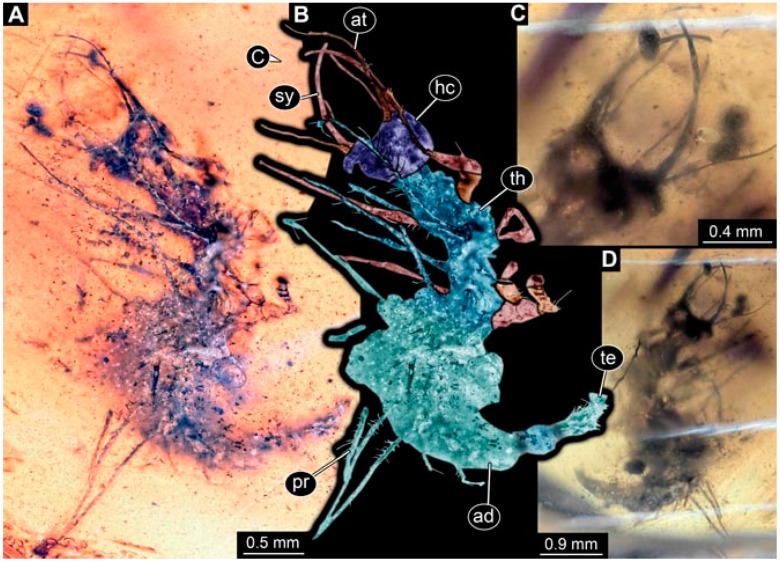
Specimen 4839 (PED 0375); Myanmar amber. (**A**) Ventral view. (**B**) Ventral view, colour-marked. (**C**) Close-up of head capsule in dorsal view. (**D**) Dorsal view. Abbreviations: ad = abdomen; at = antenna; hc = head capsule; pr = protrusions; sy = stylet; te = trunk end; th = thorax.

**Figure 19:**
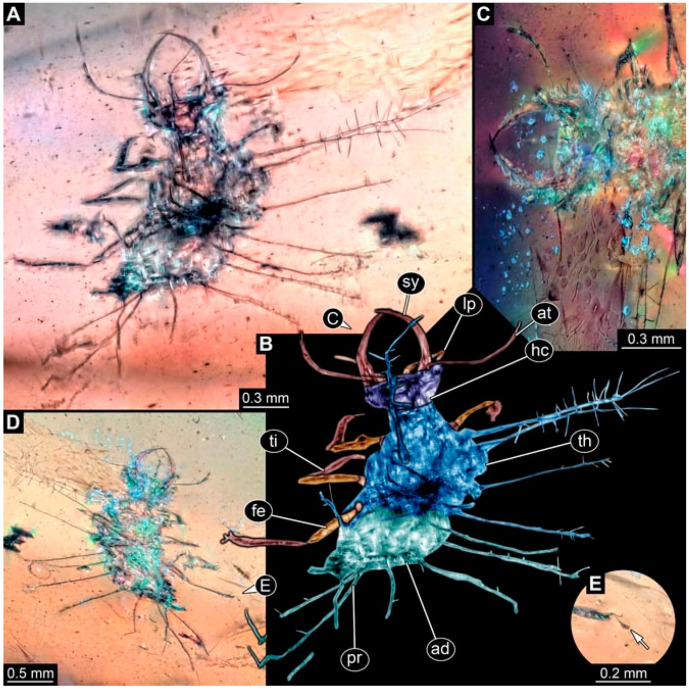
Specimen 4840 (PED 0427); Myanmar amber. (**A**) Dorsal view. (**B**) Dorsal view, colour-marked. (**C**) Close-up of head capsule in dorsal view. (**D**) Ventral view. (**E**) Close-up of distal end of trunk appendage with empodium (arrow). Abbreviations: ad = abdomen; at = antenna; fe = femur; hc = head capsule; lp = labial palp; pr = protrusions; sy = stylet; th = thorax; ti = tibia.

**Figure 20:**
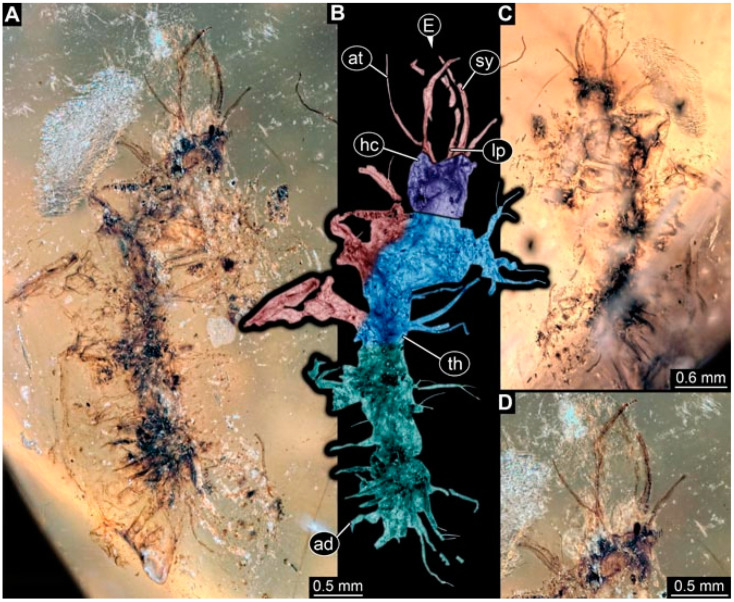
Specimen 4841 (PED 0433); Myanmar amber. (**A**) Dorsal view. (**B**) Dorsal view, colour-marked. (**C**) Ventral view. (**D**) Close-up of head capsule in dorsal view. Abbreviations: ad = abdomen; at = antenna; hc = head capsule; lp = labial palp; sy = stylet; th = thorax.

**Figure 21:**
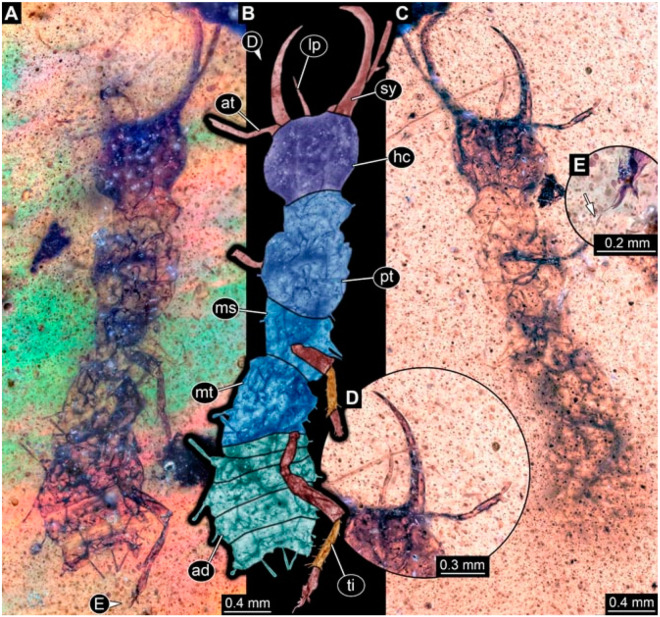
Specimen 4842 (PED 0441); Myanmar amber. (**A**) Ventral view. (**B**) Ventral view, colour-marked. (**C**) Dorsal view. (**D**) Close-up of stylet in dorsal view. (**E**) Close-up of distal end of trunk appendage with empodium (arrow). Abbreviations: ad = abdomen; at = antenna; hc = head capsule; lp = labial palp; ms = mesothorax; mt = metathorax; pt = prothorax; sy = stylet; ti = tibia.

**Figure 22:**
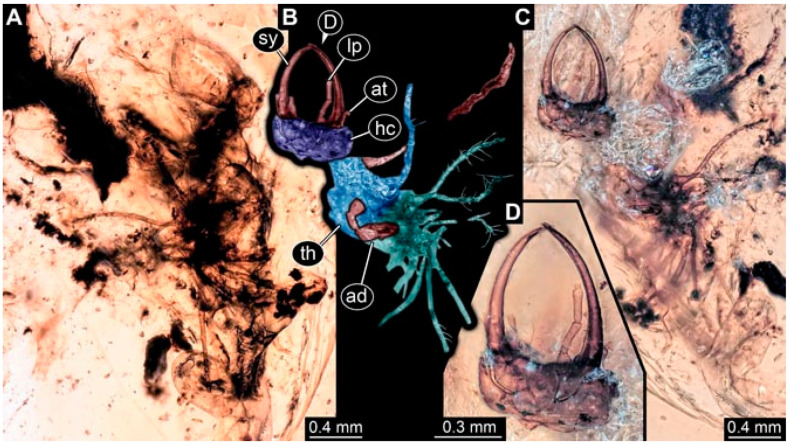
Specimen 4843 (PED 0455); Myanmar amber. (**A**) Ventral view. (**B**) Dorsal view, colour-marked. (**C**) Dorsal view. (**D**) Close-up of head capsule in dorsal view. Abbreviations: ad = abdomen; at = antenna; hc = head capsule; lp = labial palp; sy = stylet; th = thorax.

**Figure 23:**
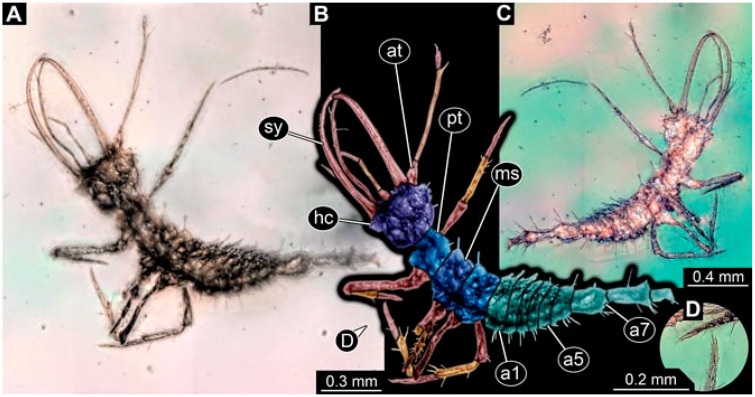
Specimen 4844 (PED 0518); Myanmar amber. (**A**) Dorsal view. (**B**) Dorsal view, colour-marked. (**C**) Ventral view. (**D**) Close-up of distal end of trunk appendage. Abbreviations: a1, 5, 7 = abdomen segments 1, 5, 7; at = antenna; hc = head capsule; ms = mesothorax; pt = prothorax; sy = stylet.

**Figure 24:**
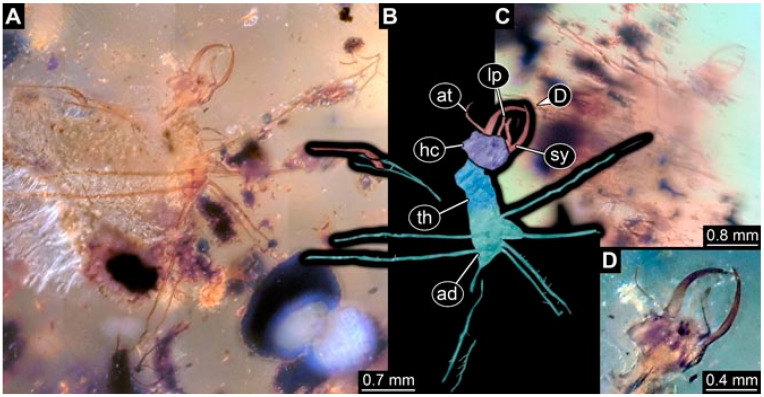
Specimen 4845 (PED 0541); Myanmar amber. (**A**) Ventral view. (**B**) Ventral view, colour-marked. (**C**) Dorsal view. (**D**) Close-up of head capsule in ventral view. Abbreviations: ad = abdomen; at = antenna; hc = head capsule; lp = labial palp; sy = stylet; th = thorax.

**Figure 25:**
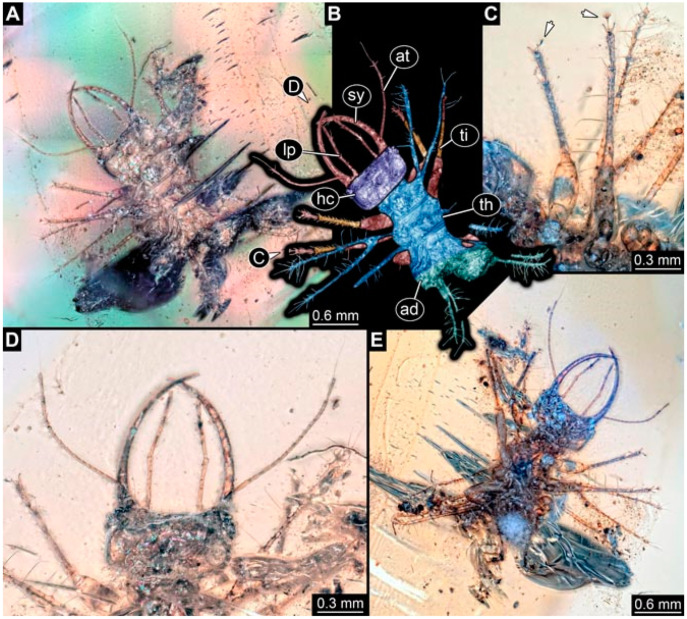
Specimen 4846 (PED 0580); Myanmar amber. (**A**) Dorsal view. (**B**) Dorsal view, colour-marked. (**C**) Close-up of trunk appendages with empodia (arrows). (**D**) Close-up of head capsule in dorsal view. (**E**) Ventral view. Abbreviations: ad = abdomen; at = antenna; hc = head capsule; lp = labial palp; sy = stylet; th = thorax; ti = tibia.

**Figure 26:**
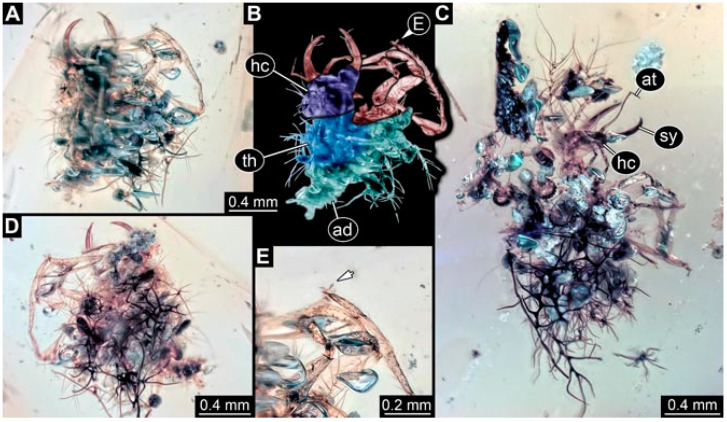
Specimen 4848 (PED 0642); Myanmar amber. (**A**) Dorsal view. (**B**) Dorsal view, colour-marked. (**C**) Ventro-lateral view. (**D**) Ventral view. (**E**) Close-up of trunk appendage with empodium (arrow). Abbreviations: ad = abdomen; at = antenna; hc = head capsule; sy = stylet; th = thorax.

**Figure 27:**
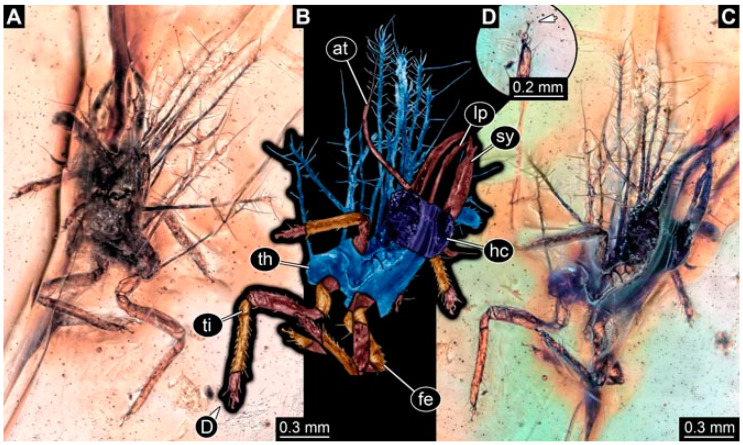
Specimen 4849 (PED 0666); Myanmar amber. (**A**) Ventral view. (**B**) Dorsal view, colour-marked. (**C**) Dorsal view. (**D**) Close-up of distal end of trunk appendage with empodium (arrow). Abbreviations: at = antenna; fe = femur; hc = head capsule; lp = labial palp; sy = stylet; th = thorax; ti = tibia.

**Figure 28:**
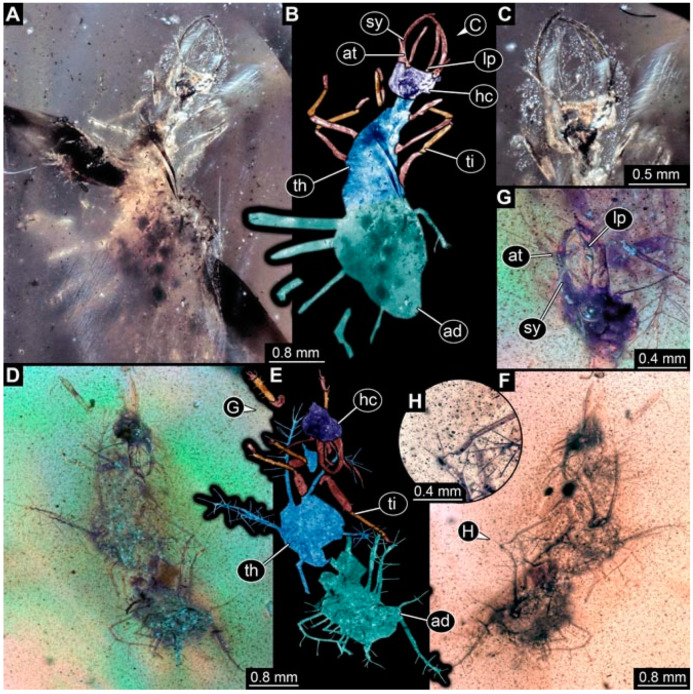
Two specimens in Myanmar amber. (**A**–**C**) Specimen 4850 (PED 0667). (**A**) Dorsal view. (**B**) Dorsal view, colour-marked. (**C**) Close-up of head capsule in dorsal view. (**D**–**H**) Specimen 4852 (PED 0715). (**D**) Ventral view. (**E**) Ventral view, colour-marked. (**F**) Dorsal view. (**G**) Close-up of head capsule in ventral view. (**H**) Close-up of distal end of trunk appendage. Abbreviations: ad = abdomen; at = antenna; hc = head capsule; lp = labial palp; sy = stylet; th = thorax; ti = tibia.

**Figure 29:**
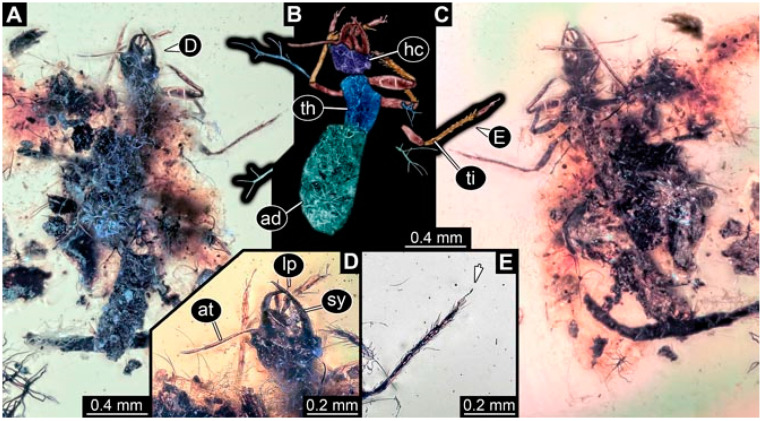
Specimen 4851 (PED 0696); Myanmar amber. (**A**) Dorsal view. (**B**) Dorsal view, colour-marked. (**C**) Ventral view. (**D**) Close-up of head capsule in dorsal view. (**E**) Close-up of trunk appendage with empodium (arrow). Abbreviations: at = antenna; ad = abdomen; hc = head capsule; lp = labial palp; sy = stylet; th = thorax; ti = tibia.

**Figure 30:**
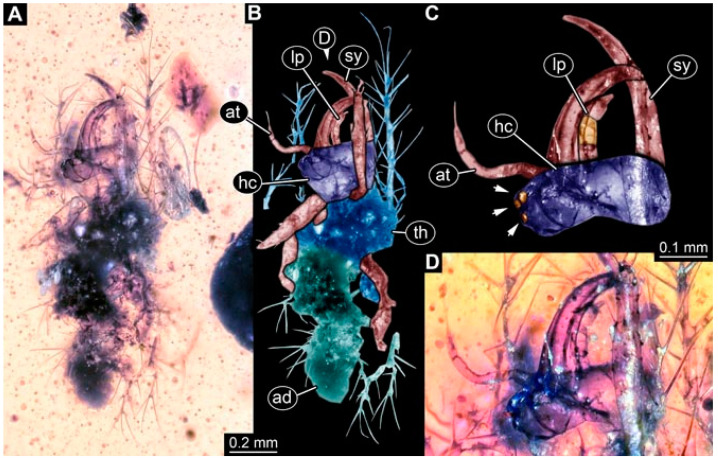
Specimen 4854 (PED 0782); Myanmar amber. (**A**) Dorsal view. (**B**) Dorsal view, colour-marked. (**C**) Close-up of head capsule in dorsal view with stemmata (arrows), colour-marked. (**D**) Close-up of head capsule in dorsal view. Abbreviations: ad = abdomen; at = antenna; hc = head capsule; lp = labial palp; sy = stylet; th = thorax.

**Figure 31:**
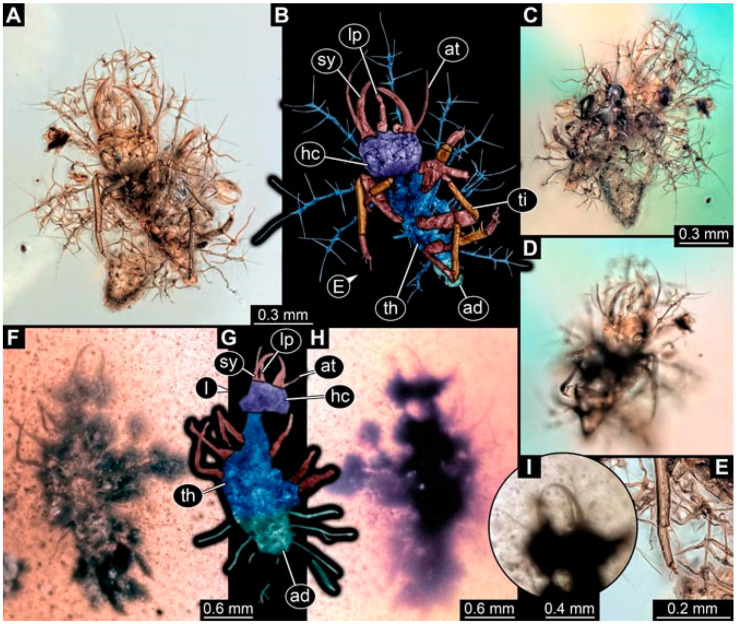
Two specimens in Myanmar amber. (**A**–**E**) Specimen 4855 (PED 0793). (**A**) Ventral view. (**B**) Ventral view, colour-marked. (**C**) Dorsal view. (**D**) Antero-dorsal view. (**E**) Close-up of trunk appendage. (**F**–**I**) Specimen 4856 (PED 0807). (**F**) Ventral view. (**G**) Ventral view, colour-marked. (**H**) Dorsal view. (**I**) Close-up of head capsule in ventral view. Abbreviations: ad = abdomen; at = antenna; hc = head capsule; lp = labial palp; sy = stylet; th = thorax; ti = tibia.

**Figure 32:**
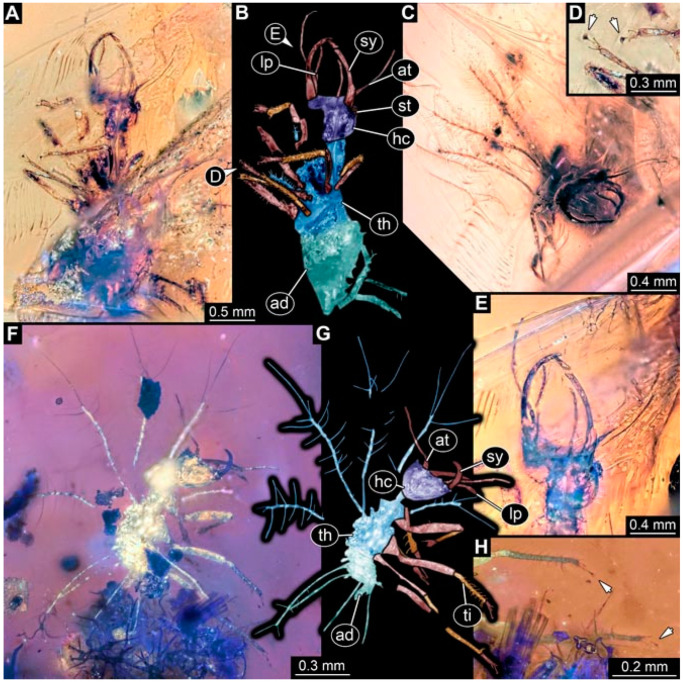
Two specimens in Myanmar amber. (**A**–**E**) Specimen 4857 (PED 0837). (**A**) Ventral view. (**B**) Ventral view, colour-marked. (**C**) Anterior view. (**D**) Close-up of trunk appendages with empodia (arrows). (**E**) Close-up of head capsule in dorsal view. (**F**–**H**) Specimen 4853 (PED 0754). (**F**) Dorsal view. (**G**) Dorsal view, colour-marked. (**H**) Close-up of trunk appendages with empodia (arrows). Abbreviations: ad = abdomen; at = antenna; hc = head capsule; lp = labial palp; st = stemmata; sy = stylet; th = thorax; ti = tibia.

**Figure 33:**
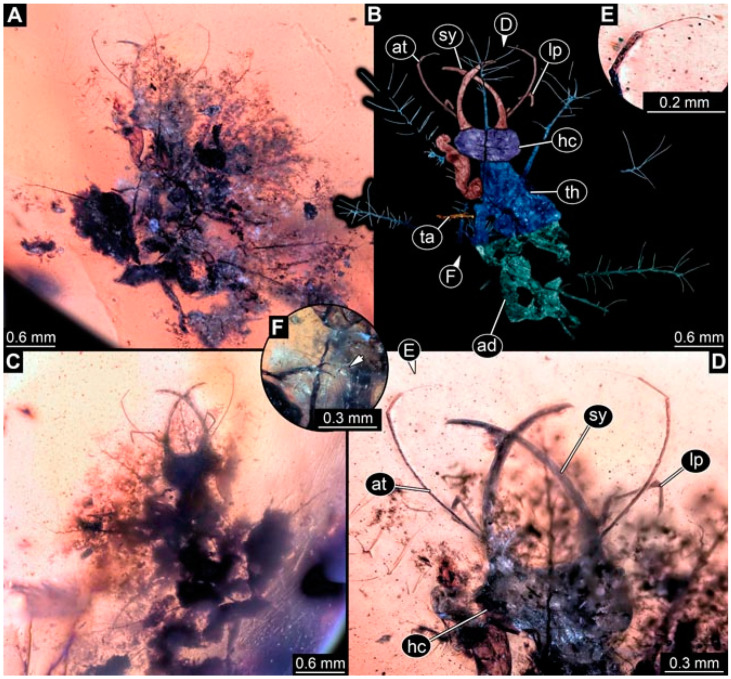
Specimen 4858 (PED 0901); Myanmar amber. (**A**) Dorsal view. (**B**) Dorsal view, colour-marked. (**C**) Ventral view. (**D**) Close-up of head capsule in dorsal view. (**E**) Close-up of antenna in dorsal view. (**F**) Close-up of trunk appendages with empodium (arrow). Abbreviations: ad = abdomen; at = antenna; hc = head capsule; lp = labial palp; sy = stylet; ta = tarsus; th = thorax.

**Figure 34:**
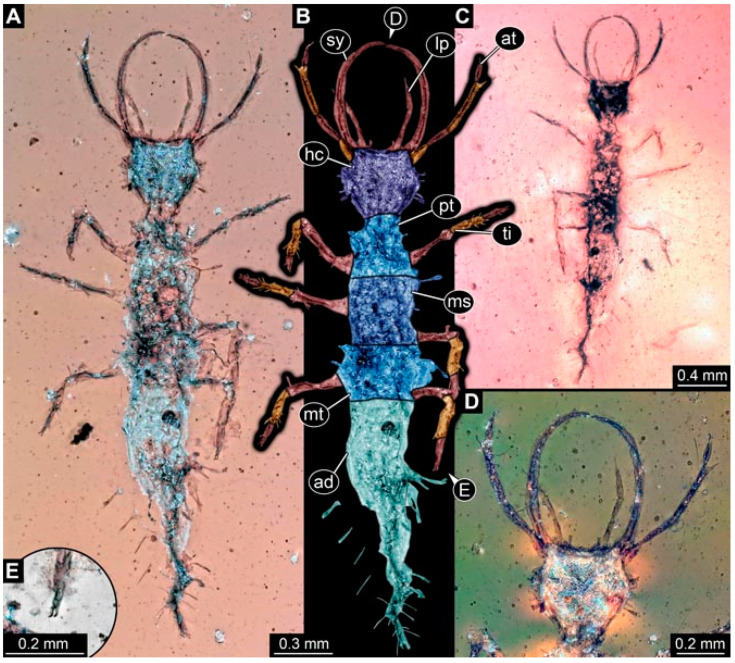
Specimen 4859 (PED 0952); Myanmar amber. (**A**) Dorsal view. (**B**) Dorsal view, colour-marked. (**C**) Ventral view. (**D**) Close-up of head capsule in dorsal view. (**E**) Close-up of distal end of trunk appendage. Abbreviations: ad = abdomen; at = antenna hc = head capsule; lp = labial palp; ms = mesothorax; mt = metathorax; pt = prothorax; sy = stylet; ti = tibia.

**Figure 35:**
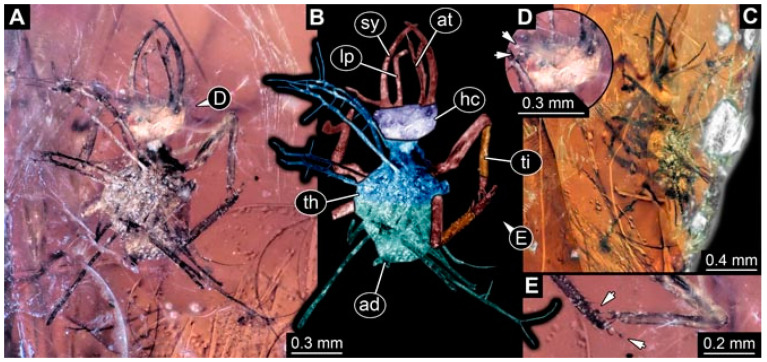
Specimen 4860 (PED 0983); Myanmar amber. (**A**) Dorsal view. (**B**) Dorsal view, colour-marked. (**C**) Ventro-lateral view. (**D**) Close-up of head capsule in dorsal view with stemmata (arrows). (**E**) Close-up of trunk appendages with empodia (arrows). Abbreviations: ad = abdomen; at = antenna; hc = head capsule; lp = labial palp; sy = stylet; th = thorax; ti = tibia.

**Figure 36:**
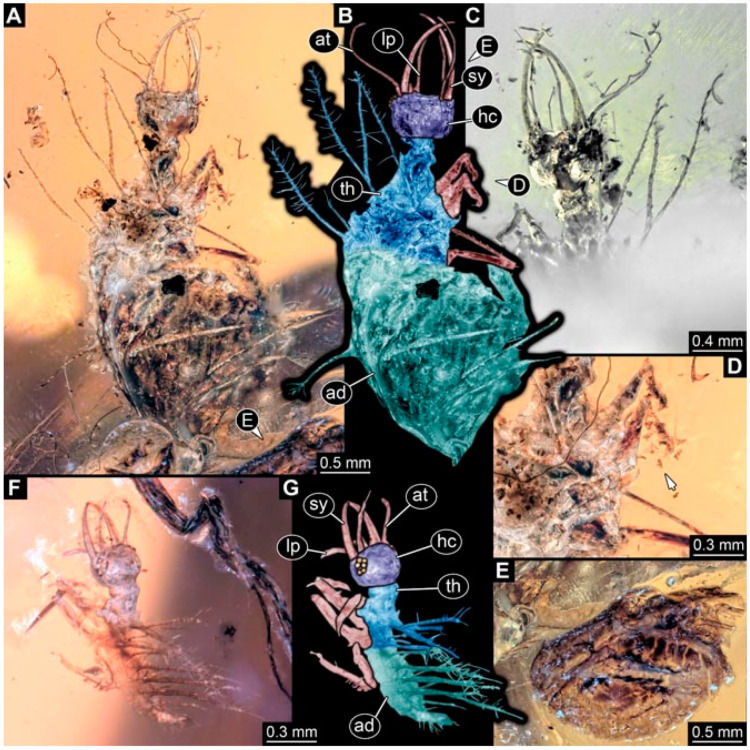
Two specimens in Myanmar amber. (**A**–**E**) Specimen 4861 (PED 0989a). (**A**) Dorsal view. (**B**) Dorsal view, colour-marked. (**C**) Close-up of head capsule in ventral view. (**D**) Close-up of trunk appendages with empodium (arrow). (**E**) Possible exuvia, or other remain. (**F**,**G**) Specimen 4862 (PED 0989b). (**F**) Dorsal view. (**G**) Dorsal view, colour-marked. Abbreviations: ad = abdomen; at = antenna; hc = head capsule; lp = labial palp; sy = stylet; th = thorax.

**Figure 37:**
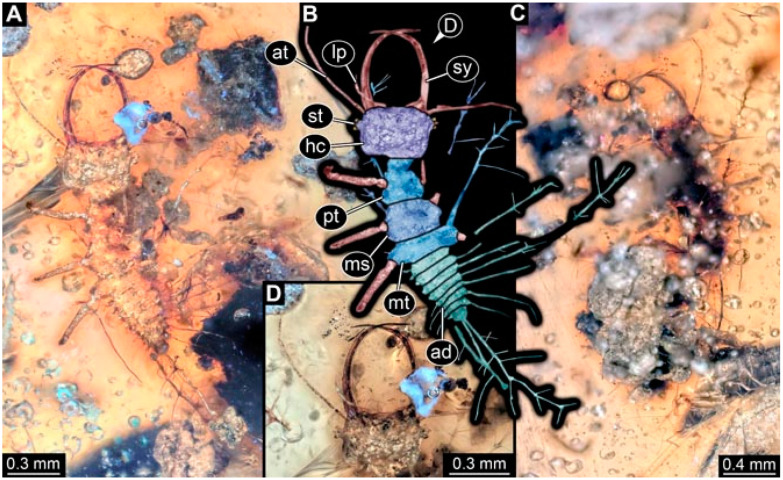
Specimen 4863 (PED 1000); Myanmar amber. (**A**) Dorsal view. (**B**) Dorsal view, colour-marked. (**C**) Ventral view. (**D**) Close-up of head capsule in dorsal view. Abbreviations: ad = abdomen; at = antenna; hc = head capsule; lp = labial palp; ms = mesothorax; mt = metathorax; pt = prothorax; st = stemmata; sy = stylet.

**Figure 38:**
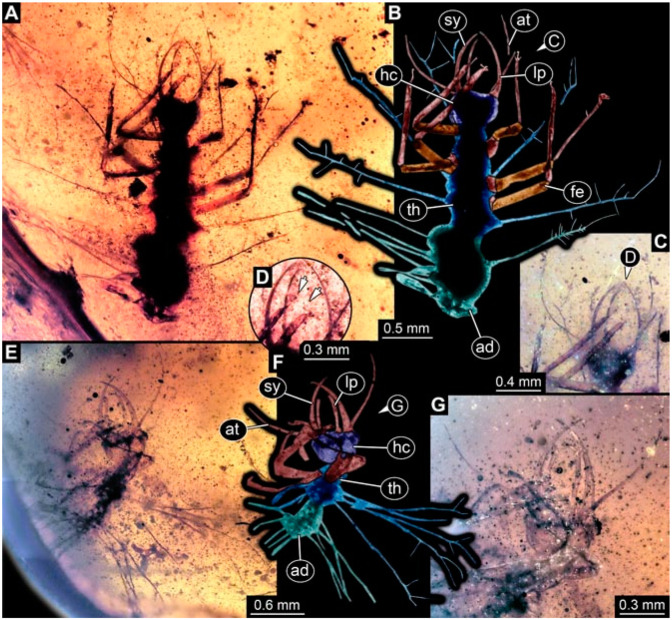
Two specimens in Myanmar amber. (**A**–**D**) Specimen 4865 (PED 1229a). (**A**) Ventral view. (**B**) Ventral view, colour-marked. (**C**) Close-up of head capsule in ventral view. (**D**) Close-up of trunk appendages with empodia (arrows). (**E**–**G**) Specimen 4866 (PED 1229b). (**E**) Ventral view. (**F**) Ventral view, colour-marked. (**G**) Close-up of head capsule in ventral view. Abbreviations: ad = abdomen; at = antenna; fe = femur; hc = head capsule; lp = labial palp; sy = stylet; th = thorax.

**Figure 39:**
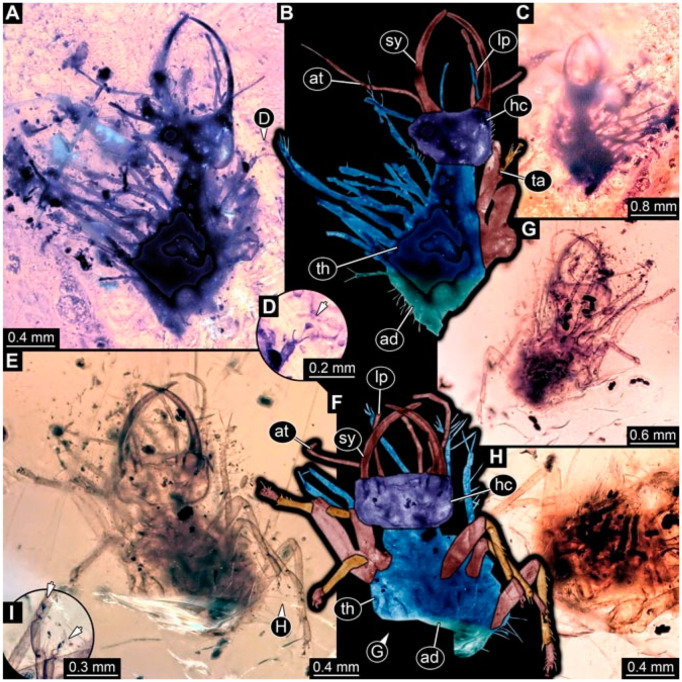
Two specimens in Myanmar amber. (**A**–**C**) Specimen 4867 (PED 1258a). (**A**) Dorsal view. (**B**) Dorsal view, colour-marked. (**C**) Ventral view. (**D**) Close-up of trunk appendage with empodium (arrow). (**E**–**I**) Specimen 4868 (PED 1258b). (**E**) Ventral view. (**F**) Ventral view, colour-marked. (**G**) Dorsal view. (**H**) Close-up of trunk in dorsal view. (**I**) Close-up of trunk appendages with empodia (arrows). Abbreviations: ad = abdomen; at = antenna; hc = head capsule; lp = labial palp; sy = stylet; ta = tarsus; th = thorax.

**Figure 40:**
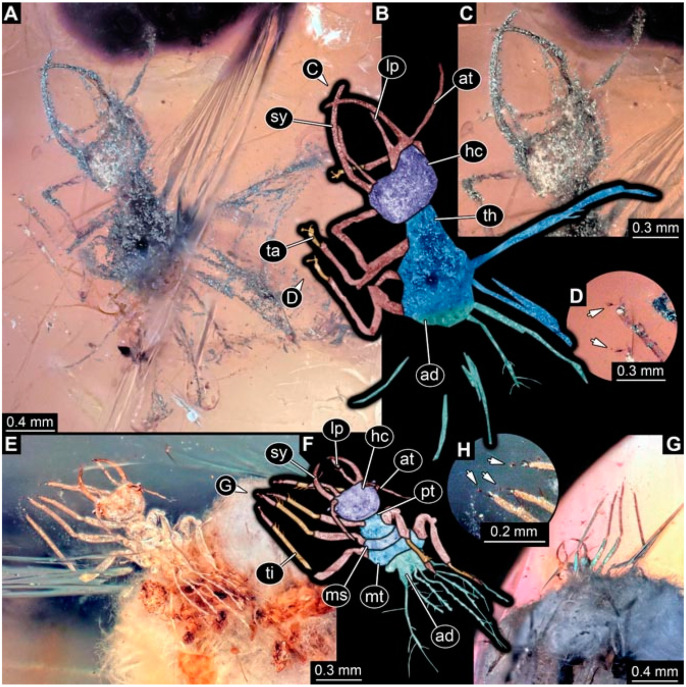
Two specimens in Myanmar amber. (**A**–**D**) Specimen 4869 (PED 1287). (**A**) Dorsal view. (**B**) Dorsal view, colour-marked. (**C**) Close-up of head capsule in dorsal view. (**D**) Close-up of trunk appendages with empodia (arrows). (**E**–**H**) Specimen 4871 (PED 1311). (**E**) Dorsal view. (**F**) Dorsal view, colour-marked. (**G**) Ventral view. (**H**) Close-up of trunk appendages with empodia (arrows). Abbreviations: ad = abdomen; at = antenna; hc = head capsule; lp = labial palp; ms = mesothorax; mt = metathorax; pt = prothorax; sy = stylet; ta = tarsus; th = thorax; ti = tibia.

**Figure 41:**
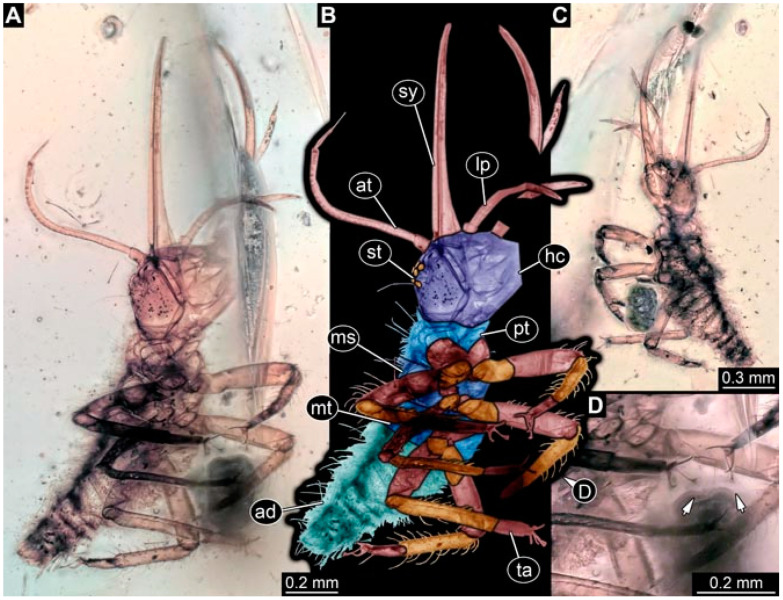
Specimen 4870 (PED 1301); Myanmar amber. (**A**) Ventral view. (**B**) Ventral view, colour-marked. (**C**) Dorso-lateral view. (**D**) Close-up of trunk appendages with empodia (arrows). Abbreviations: ad = abdomen; at = antenna; hc = head capsule; lp = labial palp; ms = mesothorax; mt = metathorax; pt = prothorax; st = stemmata; sy = stylet; ta = tarsus.

**Figure 42:**
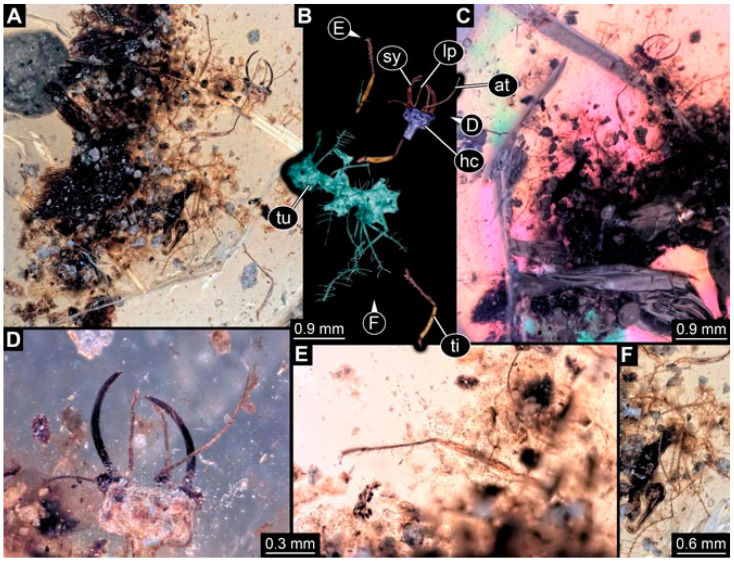
Specimen 4872 (PED 1322); Myanmar amber. (**A**) Dorsal view. (**B**) Dorsal view, colour-marked. (**C**) Ventral view. (**D**) Close-up of head capsule in dorsal view. (**E**) Close-up of trunk appendages. (**F**) Close-up of protrusions in dorsal view. Abbreviations: at = antenna; hc = head capsule; lp = labial palp; sy = stylet; ti = tibia; tu = trunk.

**Figure 43:**
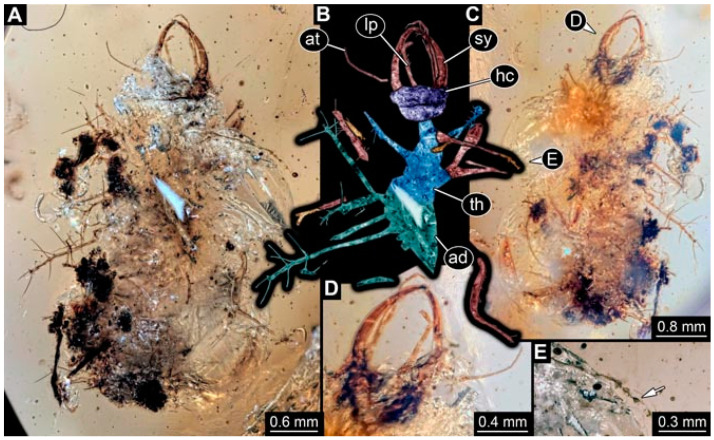
Specimen 4873 (PED 1323); Myanmar amber. (**A**) Ventral view. (**B**) Ventral view, colour-marked. (**C**) Dorsal view. (**D**) Close-up of head capsule in dorsal view. (**E**) Close-up of trunk appendage with empodium (arrow). Abbreviations: ad = abdomen; at = antenna; hc = head capsule; lp = labial palp; sy = stylet; th = thorax.

**Figure 44:**
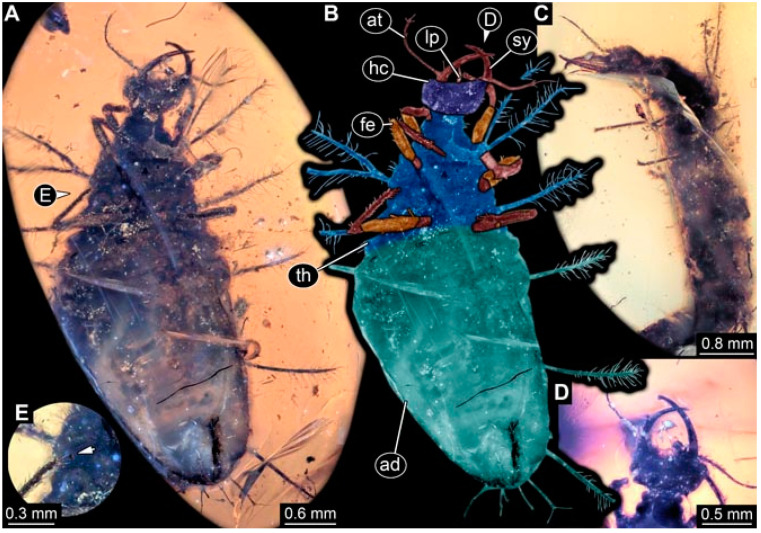
Specimen 4874 (PED 1333); Myanmar amber. (**A**) Ventral view. (**B**) Ventral view, colour-marked. (**C**) Lateral view. (**D**) Close-up of head capsule in ventral view. (**E**) Close-up of trunk appendage with empodium (arrow). Abbreviations: ad = abdomen; at = antenna; fe = femur; hc = head capsule; lp = labial palp; sy = stylet; th = thorax.

**Figure 45:**
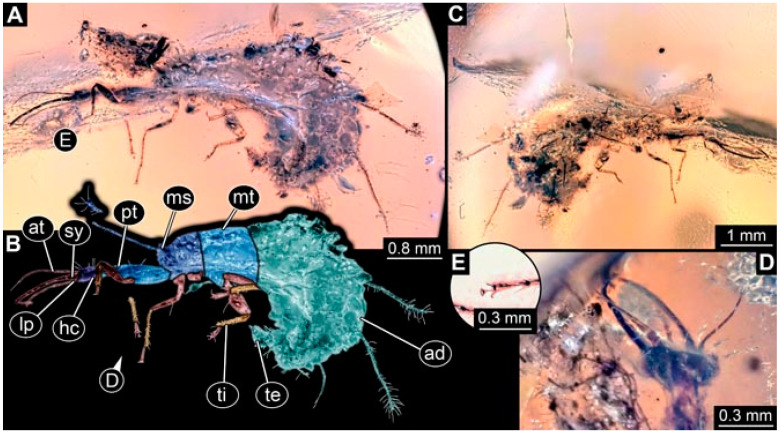
Specimen 4875 (PED 1335); Myanmar amber. (**A**) Lateral view from left side. (**B**) Lateral view from left side, colour-marked. (**C**) Lateral view from right side. (**D**) Close-up of head capsule in dorsal view. (**E**) Close-up of distal end of trunk appendage. Abbreviations: ad = abdomen; at = antenna; hc = head capsule; lp = labial palp; ms = mesothorax; mt = metathorax; pt = prothorax; sy = stylet; te = trunk end; ti = tibia.

**Figure 46:**
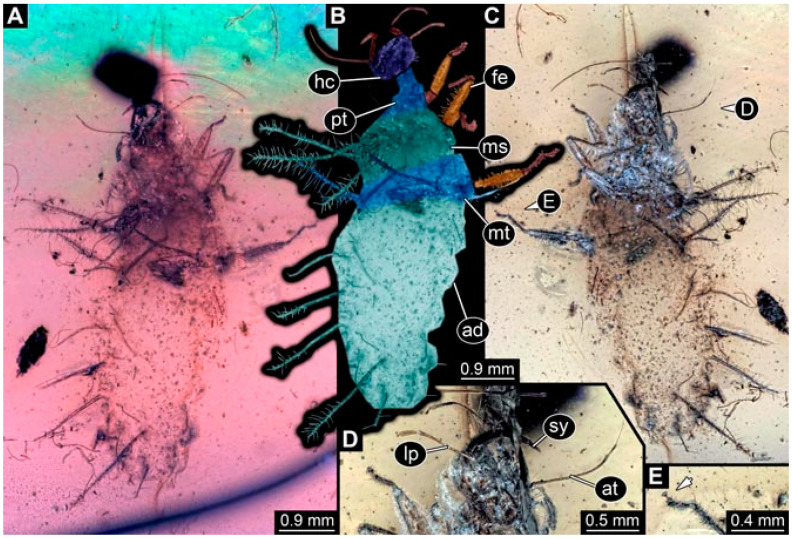
Specimen 4876 (Weiterschan BuB 11); Myanmar amber. (**A**) Dorsal view. (**B**) Dorsal view, colour-marked. (**C**) Ventral view. (**D**) Close-up of head capsule in ventral view. (**E**) Close-up of trunk appendage with empodium (arrow). Abbreviations: ad = abdomen; at = antenna; fe = femur; hc = head capsule; lp = labial palp; ms = mesothorax; mt = metathorax; pt = prothorax; sy = stylet.

**Figure 47:**
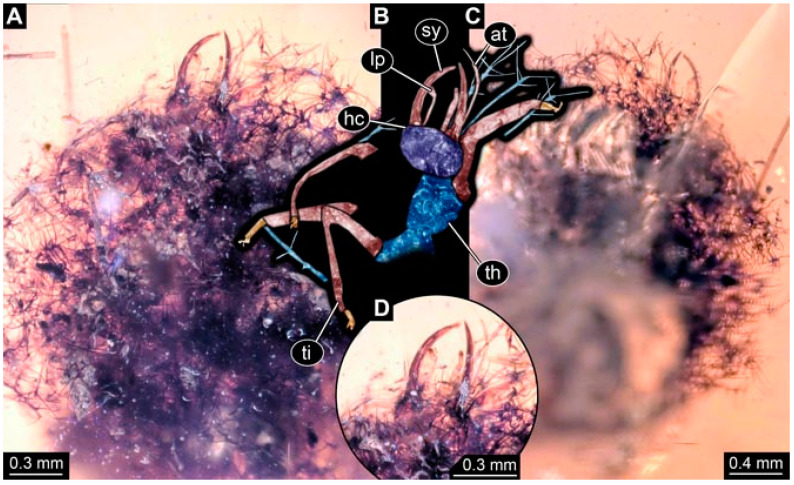
Specimen 4877 (Weiterschan BuB 31); Myanmar amber. (**A**) Dorsal view. (**B**) Dorsal view, colour-marked. (**C**) Ventral view. (**D**) Close-up of head capsule in dorsal view. Abbreviations: at = antenna; hc = head capsule; lp = labial palp; sy = stylet; th = thorax; ti = tibia.

**Figure 48:**
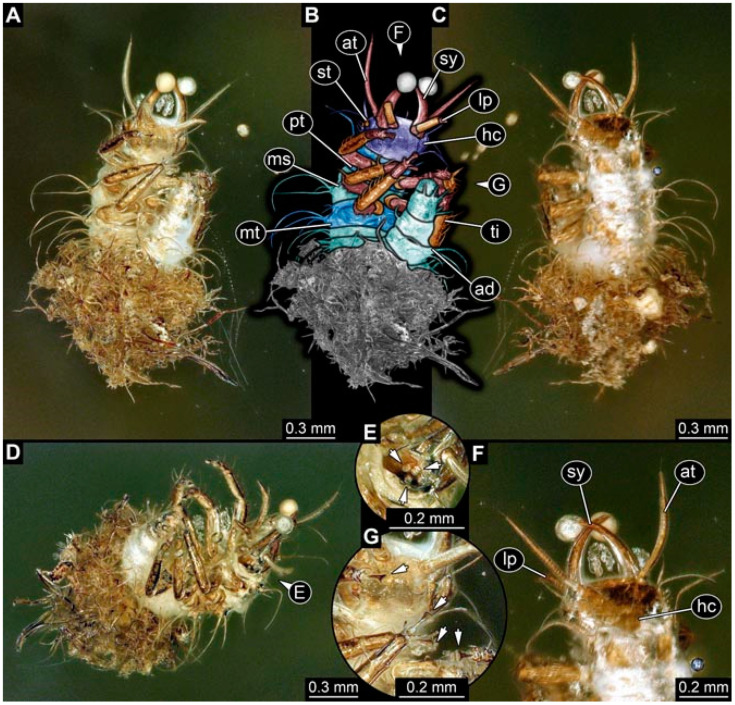
Specimen 4703 (SMF Be 2021); Chrysopidae; Baltic amber. (**A**) Ventral view. (**B**) Ventral view, colour-marked. (**C**) Dorsal view. (**D**) Antero-ventral view. (**E**) Close-up of stemmata (arrows). (**F**) Close-up of head capsule in ventral view. (**G**) Close-up of trunk appendages with empodia (arrows). Abbreviations: ad = abdomen; at = antenna; hc = head capsule; lp = labial palp; ms = mesothorax; mt = metathorax; pt = prothorax; st = stemmata; sy = stylet; ti = tibia.

**Figure 49:**
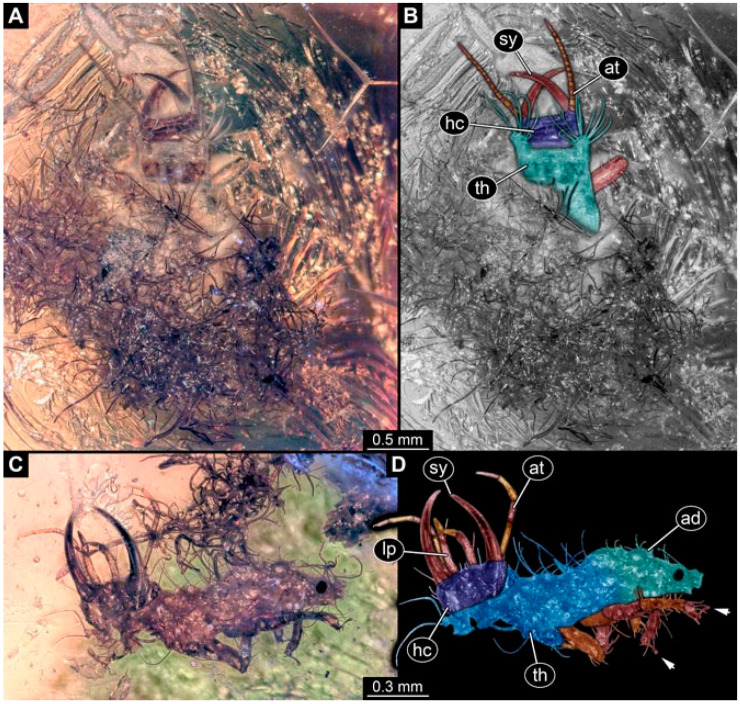
Two specimens in Baltic amber. (**A**,**B**). Specimen 4702 (CCGG 7615); Chrysopidae. (**A**) Dorsal view. (**B**) Dorsal view, colour-marked. (**C**,**D**) Specimen 4704 (SMF Be 1861); Chrysopidae. (**C**) Dorsal view. (**D**) Dorsal view, colour-marked; note trunk appendages with empodia (arrows). Abbreviations: ad = abdomen; at = antenna; hc = head capsule; lp = labial palp; sy = stylet; th = thorax.

**Figure 50:**
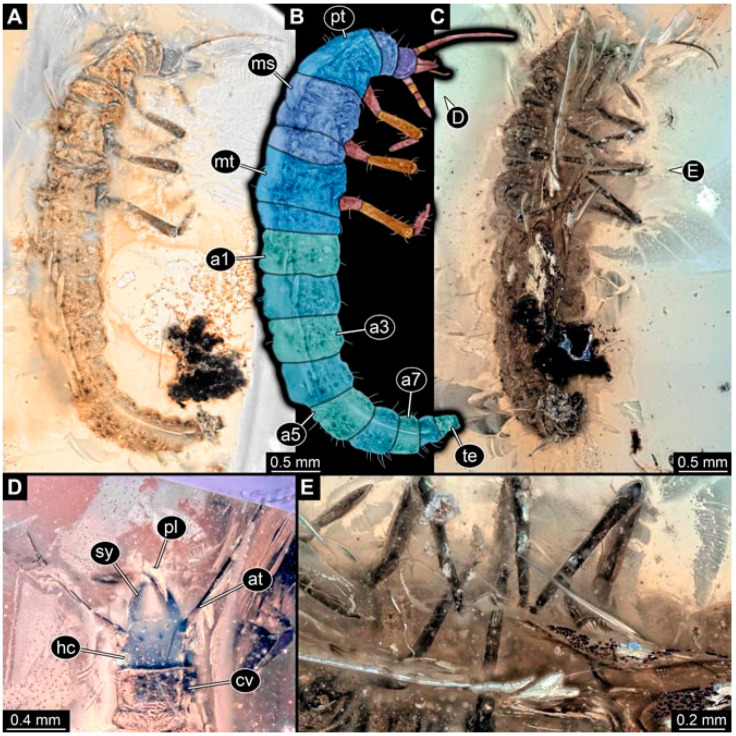
Specimen 4753 (CCHH 1786-3); Hemerobiidae; Baltic amber. (**A**) Dorsal view. (**B**) Dorsal view, colour-marked. (**C**) Ventral view. (**D**) Close-up of head capsule in dorsal view. (**E**) Close-up of trunk appendages. Abbreviations: a1–7 = abdomen segments 1–7; at = antenna; cv = cervix hc = head capsule; ms = mesothorax; mt = metathorax; pl = palp; pt = prothorax; sy = stylet; te = trunk end.

As an expanded basis for the shape analysis, numerous new aphidlions and aphidlion-like larvae are presented here. The terminology follows earlier studies [19,21,22,23,24,25]. Short descriptions of these larvae are provided in the following:

(1) Specimen 4819 (BUB 3060) is preserved in Myanmar amber. It is accessible in a dorsal (Figure 1B,C) and ventral view (Figure 1A). A prominent camouflaging cloak is present (Figure 1A). In the ventral view, the head capsule is well accessible (Figure 1F). The antenna bears a prominent seta distally (Figure 1F). Each tarsus of the anterior trunk appendages (walking legs) carries a trumpet-shaped empodium (Figure 1E). Prominent protrusions on the back are well apparent (Figure 1D). The overall length of the larva is 1.60 mm.
(2) Specimen 4821 (BUB 3066) is preserved in Myanmar amber. It is accessible in a ventral view (Figure 2A,B). No camouflaging cloak is apparent. The antenna bears a prominent seta distally (Figure 2C). Each tarsus of the trunk appendages carries a trumpet-shaped empodium (Figure 2D). Prominent protrusions on the back are well apparent (Figure 2A). The overall length of the larva is 1.69 mm.
(3) Specimen 4822 (BUB 3347) is preserved in Myanmar amber. It is accessible in a dorsal (Figure 3B,C) and ventral view (Figure 3A), yet the ventral side is less well accessible due to impurities in the amber. The head capsule is rather rectangular in its outline (Figure 3D). Traces of a camouflaging cloak are present. The antenna bears a prominent seta distally (Figure 3D). Each tarsus of the trunk appendages carries a trumpet-shaped empodium (Figure 3E). Prominent protrusions on the back are well apparent (Figure 3B,C). The overall length of the larva is 2.12 mm.
(4) Specimen 4823 (BUB 3358) is preserved in Myanmar amber. It is accessible in a dorsal (Figure 4A) and ventral view (Figure 4B,C). Parts of the body are partially separated from each other. The head shape is well apparent in dorsal view (Figure 4E). Remains of a camouflaging cloak are apparent, including an appendage of another animal. The antenna bears a prominent seta distally. Each tarsus of the trunk appendages carries a trumpet-shaped empodium (Figure 4D). Prominent protrusions on the back are well apparent (Figure 4F). The exact length cannot be measured.
(5) Specimen 4824 (BUB 3359) is preserved in Myanmar amber. It is accessible in a dorsal (Figure 5A,B) and ventral view (Figure 5C). No camouflaging cloak is apparent. The antenna bears a prominent seta distally (Figure 5D). Each tarsus of the trunk appendages carries a trumpet-shaped empodium (Figure 5F). Prominent protrusions on the back are well apparent (Figure 5E). The overall length of the larva is 1.05 mm.
(6) Specimen 4820 (BUB 3361) is preserved in Myanmar amber. It is accessible in a dorsal (Figure 6C) and ventral view (Figure 6A,B). Dorsally, the head is not well accessible, but it is ventrally (Figure 6D). No camouflaging cloak is apparent. The antenna bears a prominent seta distally (Figure 6D). Each tarsus of the trunk appendages carries a trumpet-shaped empodium (Figure 6A). Prominent protrusions on the back are apparent. The overall length of the larva is 2.56 mm.
(7) Specimen 4825 (BUB 3379) is preserved in Myanmar amber. It is accessible in a dorsal (Figure 7C) and ventral view (Figure 7A,B). No camouflaging cloak is apparent. The antenna bears a prominent, but short seta distally (Figure 7D). The trunk appendages lack empodia (Figure 7E). No prominent protrusions on the back are apparent. The overall length of the larva is 1.30 mm.
(8) Specimen 4826 (BUB 3393) is preserved in Myanmar amber. It is accessible in a dorsal (Figure 8A,B) and ventral view (Figure 8C), but is partly concealed by bubbles. The head has a roughly rectangular shape (Figure 8D). No camouflaging cloak is apparent. The antenna bears a prominent seta distally (Figure 8D). Trunk appendages are not well accessible; however, the empodia are still apparent (Figure 8E). No prominent protrusions on the back are apparent. The trunk end is not well preserved (Figure 8F). The overall length of the larva is 6.00 mm.
(9) Specimen 4827 (F 3196 BU CJW) is preserved in Myanmar amber. It is accessible in a dorsal (Figure 9A,B) and lateral view (Figure 9C). No camouflaging cloak is apparent. The stylets are relatively longer than in other specimens. The antenna bears a prominent seta distally. The trunk appendages lack empodia (Figure 9D). No prominent protrusions on the back are apparent. The overall length of the larva is 1.22 mm.
(10) Specimen 4829 (PED 0038) is preserved in Myanmar amber. It is accessible in a dorsal (Figure 9E,F) and ventral view (Figure 9G). No camouflaging cloak is apparent. The specimen is incomplete and the posterior end is missing. The antenna bears a prominent seta distally. Labial palps appear short, element 3 appears to be the longest; also, part of the proximal part of the labium appears to be apparent (Figure 9H). The trunk appendages lack empodia. No prominent protrusions on the back are apparent. The preserved part is slightly longer than 1 mm. The specimen has some characters of larvae of Hemerobiidae.
(11) Specimen 4828 (PED 0034) is preserved in Myanmar amber. It is accessible in a dorsal (Figure 10C) and ventral view (Figure 10A,B), yet the ventral view is less distorted. The thorax region is rather slender, the abdomen short, and the head is relatively large. No camouflaging cloak is apparent. The antenna bears a prominent seta distally (Figure 10D). Each tarsus of the trunk appendages carries a trumpet-shaped empodium (Figure 10E). Prominent protrusions on the back are apparent. The overall length of the larva is 2.94 mm.
(12) Specimen 4830 (PED 0065) is preserved in Myanmar amber. It is only accessible in a lateral view (Figure 11A–C), hence the head and stylet shape are not accessible. No camouflaging cloak is apparent (Figure 11A–C). The antenna bears a prominent seta distally. Each tarsus of the trunk appendages carries a trumpet-shaped empodium (Figure 11D). Prominent protrusions on the back are apparent (Figure 11A–C). The trunk end is not well accessible; therefore, the total length of the specimen cannot be measured.
(13) Specimen 4832 (PED 0248) is preserved in Myanmar amber. It is accessible in a dorsal (Figure 11E,F) and ventral view (Figure 11G). No camouflaging cloak is apparent. The antennae and labial palps are rather short and broad (Figure 11H). The antenna does not bear a prominent seta distally. The trunk appendages lack empodia (Figure 11I). No prominent protrusions on the back are apparent. The overall length of the larva is 1.12 mm, but the trunk end is partly enrolled ventrally (Figure 11J); the true length should have been slightly longer. The specimen has some characters of larvae of Hemerobiidae.
(14) Specimen 4831 (PED 0149) is preserved in Myanmar amber. It is accessible in a dorsal (Figure 12A,B) and ventral view (Figure 12C). No camouflaging cloak is apparent. It has labial palps with three elements; the terminal one is the longest (Figure 12D,E). The antenna bears a prominent seta distally. No tarsi are accessible; hence, it remains unclear whether they bear empodia. No prominent protrusions on the back are apparent. The overall length of the larva is 6.19 mm. The specimen has some characters of larvae of Hemerobiidae.
(15) Specimen 4833 (PED 0251) is preserved in Myanmar amber. It is accessible in a dorsal (Figure 13A,B) and ventral view (Figure 13D). No camouflaging cloak is apparent. The antenna bears a prominent seta distally (Figure 13C). Each tarsus of the trunk appendages carries a trumpet-shaped empodium (Figure 13C). Prominent protrusions on the back are apparent. The overall length of the larva is 3.37 mm.
(16) Specimen 4834 (PED 0252) is preserved in Myanmar amber. It is accessible in a dorsal to dorso-lateral (Figure 14A,B) and ventral view (Figure 14C). No camouflaging cloak is apparent. The antenna bears a prominent seta distally. Each tarsus of the trunk appendages carries a trumpet-shaped empodium (Figure 14D). Prominent protrusions on the back are apparent and distally bear small objects. The overall length of the larva is 3.79 mm.
(17) Specimen 4835 (PED 0253) is preserved in Myanmar amber. It is accessible in a dorsal (Figure 15A,B) and ventral view (Figure 15C). No camouflaging cloak is apparent. Head and head appendages appear rather large in comparison to the trunk. The antenna bears a prominent seta distally. The labial palps have three elements; the second is the longest (Figure 15D). The trunk appendages lack empodia. No prominent protrusions on the back are apparent. The overall length of the larva is 1.05 mm.
(18) Specimen 4836 (PED 0315) is preserved in Myanmar amber. It is accessible in a dorsal (Figure 16A,B) and ventral view (Figure 16C). A prominent camouflaging cloak is present, partly concealing the dorsal side. The antenna bears a prominent seta distally. Each tarsus of the trunk appendages carries a trumpet-shaped empodium (Figure 16D). Prominent protrusions on the back are apparent. The overall length of the larva is 2.31 mm.
(19) Specimen 4837 (PED 0323) is preserved in Myanmar amber. It is accessible in a dorsal (Figure 16E,F) and ventral view (Figure 16G). No camouflaging cloak is apparent. The antenna bears a prominent seta distally. Each tarsus of the trunk appendages carries a trumpet-shaped empodium (Figure 16H). Prominent protrusions on the back are apparent. The abdomen is rather short. The overall length of the larva is 0.61 mm.
(20) Specimen 4838 (PED 0330) is preserved in Myanmar amber. It is accessible in a dorsal (Figure 17A,B) and ventral view, yet the ventral view is partly concealed by impurities of the amber (Figure 17D). No camouflaging cloak is apparent. The tips of the antennae are not accessible (Figure 17C). Each tarsus of the trunk appendages carries a trumpet-shaped empodium. Prominent protrusions on the back are apparent. The overall length of the larva is 2.69 mm.
(21) Specimen 4839 (PED 0375) is preserved in Myanmar amber. It is accessible in a dorsal (Figure 18D) and ventral view, yet the ventral view is partly concealed by impurities of the amber (Figure 18A,B). A prominent camouflaging cloak is present. The antenna bears a prominent seta distally (Figure 18C). Each tarsus of the trunk appendages carries a trumpet-shaped empodium. Prominent protrusions on the back are apparent. The overall length of the larva is 3.87 mm.
(22) Specimen 4840 (PED 0427) is preserved in Myanmar amber. It is accessible in a dorsal (Figure 19A,B) and ventral view (Figure 19D), yet the general view is partly concealed by impurities of the amber. A prominent camouflaging cloak is present. The antenna bears a prominent seta distally (Figure 19C). Each tarsus of the trunk appendages carries a trumpet-shaped empodium (Figure 19E). Prominent protrusions on the back are apparent. The overall length of the larva is 1.65 mm.
(23) Specimen 4841 (PED 0433) is preserved in Myanmar amber. It is accessible in a dorsal (Figure 20A,B) and ventral view, yet the ventral view is partly concealed by impurities of the amber (Figure 20C). In general, the surface of the animal is not well accessible, and many details cannot be clearly recognised. No camouflaging cloak is apparent. The antenna bears a prominent seta distally; it is almost as long as the main antenna itself (Figure 20D). No tarsi are accessible; hence, it remains unclear if they bear empodia. Prominent protrusions on the back are apparent. The overall length of the larva is 3.73 mm.
(24) Specimen 4842 (PED 0441) is preserved in Myanmar amber. It is accessible in a dorsal (Figure 21C) and ventral view, yet ventrally several bubbles conceal many details (Figure 21A,B). The antenna bears a prominent, but short seta distally (Figure 21D). Each tarsus of the trunk appendages carries a trumpet-shaped empodium (Figure 21E). Thorax segments appear rather elongated in comparison to the abdomen. No prominent protrusions on the back are apparent. The overall length of the larva is 3.30 mm.
(25) Specimen 4843 (PED 0455) is preserved in Myanmar amber. It is accessible in a dorsal (Figure 22B,C) and ventral view (Figure 22A), but many details are concealed by impurities in the amber. No camouflaging cloak is apparent. The head is well accessible in the dorsal view, but the antennae and labial palps are incomplete (Figure 22D). No tarsi are accessible; hence, it remains unclear if they bear empodia. Prominent protrusions on the back are apparent. The overall length of the larva is 1.44 mm.
(26) Specimen 4844 (PED 0518) is preserved in Myanmar amber. It is accessible in a dorsal (Figure 23A,B) and ventral view (Figure 23C). No camouflaging cloak is apparent. The antenna bears a prominent seta distally. The trunk appendages lack empodia (Figure 23D). No prominent protrusions on the back are apparent. The overall length of the larva is 1.62 mm.
(27) Specimen 4845 (PED 0541) is preserved in Myanmar amber. It is accessible in dorsal (Figure 24C) and ventral view (Figure 24A,B), but details are often concealed by impurities of the amber. No camouflaging cloak is apparent. The antenna bears a prominent seta distally (Figure 24D). No tarsi are accessible; hence, it remains unclear if they bear empodia. Prominent protrusions on the back are apparent. The overall length of the larva is 2.01 mm.
(28) Specimen 4846 (PED 0580) is preserved in Myanmar amber. It is accessible in a dorsal (Figure 25A,B) and ventral view (Figure 25E), but details are often concealed by impurities of the amber. A prominent camouflaging cloak is present. The head is roughly rectangular (Figure 25D). The antenna bears a prominent seta distally (Figure 25D). Each tarsus of the trunk appendages carries a trumpet-shaped empodium (Figure 25C). Prominent protrusions on the back are apparent. The overall length of the larva is 1.89 mm.
(29) Specimen 4848 (PED 0642) is preserved in Myanmar amber. It is accessible in a dorsal (Figure 26A,B), ventral (Figure 26D), and ventro-lateral view (Figure 26C). The head appears partly disarticulated. A prominent camouflaging cloak is present. The antenna bears a prominent seta distally. Each tarsus of the trunk appendages carries a trumpet-shaped empodium (Figure 26E). Prominent protrusions on the back are apparent. The exact length cannot be estimated due to the camouflaging cloak.
(30) Specimen 4849 (PED 0666) is preserved in Myanmar amber. It is accessible in a dorsal (Figure 27B,C) and ventral view (Figure 27A), the head only in dorsal view. No camouflaging cloak is apparent. The antenna bears a prominent seta distally. Each tarsus of the trunk appendages carries a trumpet-shaped empodium (Figure 27D). Prominent protrusions on the back are apparent. The trunk appears damaged; the length can therefore not be measured.
(31) Specimen 4850 (PED 0667) is preserved in Myanmar amber. It is only accessible in dorsal view (Figure 28A,B). No camouflaging cloak is apparent. The head is roughly rectangular (Figure 28C). The antennae are not accessible. No tarsi are accessible; hence, it remains unclear if they bear empodia. Prominent protrusions on the back are apparent. The overall length of the larva is 3.61 mm.
(32) Specimen 4851 (PED 0696) is preserved in Myanmar amber. It is accessible in a dorsal (Figure 29A,B) and ventral view (Figure 29C). A prominent camouflaging cloak is present. The antenna bears a prominent seta distally. The labial palps appear rather broad (Figure 29D). Each tarsus of the trunk appendages carries a trumpet-shaped empodium (Figure 29E). Prominent protrusions on the back are apparent. The exact length cannot be measured.
(33) Specimen 4852 (PED 0715) is preserved in Myanmar amber. It is accessible in a dorsal (Figure 28F) and ventral view (Figure 28D,E). The head is separated from the trunk (Figure 28G). Many details of the trunk region are concealed by numerous bubbles. No clear camouflaging cloak is apparent. The antenna bears a prominent seta distally. Each tarsus of the trunk appendages carries a trumpet-shaped empodium (Figure 28H). Prominent protrusions on the back are apparent. The exact length cannot be measured.
(34) Specimen 4854 (PED 0782) is preserved in Myanmar amber. It is only accessible in dorsal view (Figure 30A,B). No clear camouflaging cloak is apparent. The antenna bears a prominent seta distally (Figure 30C,D). Each tarsus of the trunk appendages carries a trumpet-shaped empodium. Prominent long protrusions on the back are apparent. The overall length of the larva is 0.95 mm.
(35) Specimen 4855 (PED 0793) is preserved in Myanmar amber. It is accessible in a dorsal (Figure 31C,D) and ventral view (Figure 31A,B). A prominent camouflaging cloak is present, concealing many details of the animal. The antenna bears a prominent seta distally. Each tarsus of the trunk appendages carries a trumpet-shaped empodium (Figure 31E). Prominent protrusions on the back are apparent. The overall length of the larva is 0.87 mm.
(36) Specimen 4856 (PED 0807) is preserved in Myanmar amber. It is accessible in a dorsal (Figure 31H) and ventral view (Figure 31F,G). A prominent camouflaging cloak is present. The head is roughly rectangular (Figure 31I). The antenna bears a prominent seta distally. Each tarsus of the trunk appendages carries a trumpet-shaped empodium. Prominent protrusions on the back are apparent. The overall length of the larva is 2.16 mm.
(37) Specimen 4853 (PED 0754) is preserved in Myanmar amber. It is only accessible in dorsal view (Figure 32F,G). No clear camouflaging cloak is apparent. The antenna bears a prominent seta distally. Each tarsus of the trunk appendages carries a trumpet-shaped empodium (Figure 32H). Prominent long protrusions on the back are apparent. The overall length of the larva is 0.82 mm.
(38) Specimen 4857 (PED 0837) is preserved in Myanmar amber. It is accessible in a ventral (Figure 32A,B) and anterior view (Figure 32C), yet the ventral view is partly concealed by impurities of the amber. The head is partly damaged (Figure 32E). No camouflaging cloak is apparent. The antenna bears a prominent seta distally. Each tarsus of the trunk appendages carries a trumpet-shaped empodium (Figure 32D). Prominent protrusions on the back are apparent. The overall length of the larva is 2.40 mm.
(39) Specimen 4858 (PED 0901) is preserved in Myanmar amber. It is accessible in a dorsal (Figure 33A,B) and ventral view (Figure 33C). A prominent camouflaging cloak is present. The head is rather short and broad (Figure 33D). The antenna bears a prominent seta distally (Figure 33E). Each tarsus of the trunk appendages carries a trumpet-shaped empodium (Figure 33F). Prominent protrusions on the back are apparent. The exact length cannot be measured.
(40) Specimen 4859 (PED 0952) is preserved in Myanmar amber. It is accessible in a dorsal (Figure 34A,B) and ventral view (Figure 34C), but is partly concealed by bubbles. No camouflaging cloak is apparent. The antenna bears a prominent, but short seta distally (Figure 34D). The trunk appendages, bear claws, but lack empodia (Figure 34E). Prominent, but short protrusions on the back are apparent. The overall length of the larva is 2.53 mm.
(41) Specimen 4860 (PED 0983) is preserved in Myanmar amber. It is accessible in a dorsal (Figure 35A,B) and lateral to ventro-lateral view (Figure 35C). A prominent camouflaging cloak is present. The antenna bears a prominent seta distally (Figure 35D). Each tarsus of the trunk appendages carries a trumpet-shaped empodium (Figure 35E). Prominent long protrusions on the back are apparent. The overall length of the larva is 0.98 mm.
(42) Specimen 4861 (PED 0989a) is preserved in Myanmar amber. It is accessible in a dorsal (Figure 36A,B) and ventral view (Figure 36C), dorsally the posterior trunk is concealed (Figure 36E), ventrally only the head and part of the thorax are accessible. A prominent camouflaging cloak is present. The head is roughly rectangular (Figure 36C). The antenna has no prominent seta distally. No tarsi are accessible; hence, it remains unclear if they bear empodia. Each tarsus of the trunk appendages carries a trumpet-shaped empodium (Figure 36D). Prominent protrusions on the back are apparent. The overall length of the larva is 3.32 mm.
(43) Specimen 4862 (PED 0989b) is preserved in Myanmar amber, in the same amber piece as PED 0989a. It is accessible only in a dorso-lateral view (Figure 36F,G). No camouflaging cloak is apparent. The head bears stemmata. The antenna bears a prominent seta distally. Each tarsus of the trunk appendages carries a trumpet-shaped empodium. Prominent protrusions on the back are apparent. The overall length of the larva is 1.23 mm.
(44) Specimen 4863 (PED 1000) is preserved in Myanmar amber. It is accessible in a dorsal (Figure 37A,B) and ventral view (Figure 37C), yet dorsally some structures conceal certain aspects. No camouflaging cloak is apparent. The head is rectangular (Figure 37D). The distal tips of the antennae are not well accessible. No tarsi are accessible; hence, it remains unclear if they bear empodia. Prominent protrusions on the back are apparent. The overall length of the larva is 1.29 mm.
(45) Specimen 4864 (PED 1223) is preserved in Myanmar amber. It is accessible in a dorsal (Figure 9K) and ventral view (Figure 9I,J). A whitish structure conceals part of the thorax in the ventral view (Figure 9I,J). No camouflaging cloak is apparent. The antenna bears no prominent seta distally (Figure 9L). The trunk appendages lack empodia (Figure 9M). No prominent protrusions on the back are apparent. The overall length of the larva is 0.88 mm.
(46) Specimen 4865 (PED 1229a) is preserved in Myanmar amber. It is accessible only in a ventral view (Figure 38A,B). No camouflaging cloak is apparent. The antenna bears a prominent seta distally (Figure 38C). Each tarsus of the trunk appendages carries a trumpet-shaped empodium (Figure 38D). Prominent long protrusions on the back are apparent. The overall length of the larva is 2.34 mm.
(47) Specimen 4866 (PED 1229b) is preserved in Myanmar amber, in the same amber piece as PED 1229a. It is only accessible in ventral view (Figure 38E,F). The thorax and abdomen are partly deformed. It remains partly unclear whether a camouflaging cloak is present. The antenna bears a prominent seta distally (Figure 38G). Each tarsus of the trunk appendages carries a trumpet-shaped empodium. Prominent long protrusions on the back are apparent. The overall length of the larva is 1.24 mm.
(48) Specimen 4867 (PED 1258a) is preserved in Myanmar amber. It is accessible in a dorsal (Figure 39A,B) and ventral view (Figure 39C). A prominent camouflaging cloak is present. The antenna bears a prominent seta distally. Each tarsus of the trunk appendages carries a trumpet-shaped empodium (Figure 39D). Prominent protrusions on the back are apparent. The overall length of the larva is 1.78 mm.
(49) Specimen 4868 (PED 1258b) is preserved in Myanmar amber, in the same amber piece as PED 1258a. It is accessible in a dorsal (Figure 39G) and ventral view (Figure 39E,F). A prominent camouflaging cloak is present, but more towards the posterior (Figure 39G,H). The antenna bears a prominent seta distally. Each tarsus of the trunk appendages carries a trumpet-shaped empodium (Figure 39I). Prominent protrusions on the back are apparent. The overall length of the larva is 1.94 mm.
(50) Specimen 4869 (PED 1287) is preserved in Myanmar amber. It is only accessible in a dorsal view (Figure 40A,B). Many bubbles surround the animal, making it unclear whether a camouflaging cloak is present. The distal tip of the antenna is not accessible (Figure 40C). Each tarsus of the trunk appendages carries a trumpet-shaped empodium (Figure 40D). Prominent protrusions on the back are apparent. The overall length of the larva is 1.37 mm.
(51) Specimen 4870 (PED 1301) is preserved in Myanmar amber. It is accessible in a dorso-lateral (Figure 41C) and ventral view (Figure 41A,B). The stylets are quite long, about as long as the trunk. No camouflaging cloak is apparent. The antenna bears a prominent seta distally. Each tarsus of the trunk appendages carries a trumpet-shaped empodium (Figure 41D). No prominent protrusions on the back are apparent, but there are setae along the trunk. The overall length of the larva is 1.45 mm.
(52) Specimen 4871 (PED 1311) is preserved in Myanmar amber. It is accessible in a dorsal (Figure 40E,F) and ventral view (Figure 40G), but is ventrally less well accessible. Some material at the posterior end may be the remains of a camouflaging cloak, yet this remains unclear. The antenna bears a prominent seta distally. Each tarsus of the trunk appendages carries a trumpet-shaped empodium (Figure 40H). Prominent protrusions on the back are apparent. The exact length cannot be measured.
(53) Specimen 4872 (PED 1322) is preserved in Myanmar amber. It is accessible in a dorsal (Figure 42A,B) and ventral view (Figure 42C). The trunk is separated into several pieces and spread through the amber piece. A prominent camouflaging cloak is present. The antenna bears a prominent seta distally (Figure 42D). Each tarsus of the trunk appendages carries a trumpet-shaped empodium (Figure 42E). Prominent protrusions on the back are apparent (Figure 42F). The exact length cannot be measured.
(54) Specimen 4873 (PED 1323) is preserved in Myanmar amber. It is accessible in a dorsal (Figure 43C,D) and ventral view (Figure 43A,B), but is ventrally partly concealed by impurities in the amber. A prominent camouflaging cloak is present. The antenna bears a prominent seta distally. Each tarsus of the trunk appendages carries a trumpet-shaped empodium (Figure 43E). Prominent protrusions on the back are apparent. The overall length of the larva is 2.37 mm.
(55) Specimen 4874 (PED 1333) is preserved in Myanmar amber. It is accessible in a ventral (Figure 44A,B,D) and lateral view (Figure 44C). A prominent camouflaging cloak is present. The antenna bears a prominent seta distally. Each tarsus of the trunk appendages carries a trumpet-shaped empodium (Figure 44E). Prominent protrusions on the back are apparent. The overall length of the larva is 4.91 mm.
(56) Specimen 4875 (PED 1335) is preserved in Myanmar amber. It is accessible in a lateral (Figure 45A–C) and partly in a dorsal view; the head, especially, is accessible in dorsal view (Figure 45D). A prominent camouflaging cloak is present. The antenna bears a prominent seta distally. Each tarsus of the trunk appendages carries a trumpet-shaped empodium (Figure 45E). Prominent protrusions on the back are apparent. The overall length of the larva is 4.73 mm.
(57) Specimen 4876 (Weiterschan BuB 11) is preserved in Myanmar amber. It is accessible in a dorsal (Figure 46A,B) and ventral view (Figure 46C). A darker object conceals part of the head appendages (Figure 46D). No camouflaging cloak is apparent. The antenna bears a prominent seta distally (Figure 46D). Each tarsus of the trunk appendages carries a trumpet-shaped empodium (Figure 46E). Prominent protrusions on the back are apparent. The overall length of the larva is 5.79 mm.
(58) Specimen 4877 (Weiterschan BuB 31) is preserved in Myanmar amber. It is accessible in a dorsal (Figure 47A,B) and ventral view (Figure 47C). A prominent camouflaging cloak is present, largely concealing details of the animal. The antenna bears a prominent seta distally (Figure 47D). Each tarsus of the trunk appendages carries a trumpet-shaped empodium. Prominent protrusions on the back are apparent. The exact length cannot be measured.
(59) Specimen 4703 (SMF Be 2021) is preserved in Baltic amber. It is accessible in a dorsal (Figure 48C), ventral (Figure 48A,B) and antero-ventral view (Figure 48D,E). Two round objects conceal the tips of the stylets ventrally. A prominent camouflaging cloak is present. The antenna bears a prominent seta distally (Figure 48F). Each tarsus of the trunk appendages carries a trumpet-shaped empodium (Figure 48G). Prominent protrusions on the back are apparent. The overall length of the larva is 1.66 mm.
(60) Specimen 4702 (CCGG 7615) is preserved in Baltic amber. It is accessible in a dorsal view (Figure 49A,B). A prominent camouflaging cloak is present, largely concealing details of the animals. The head is rectangular. The antenna bears no prominent seta distally. No tarsi are accessible; hence, it remains unclear if they bear empodia. Prominent protrusions on the back are apparent. The exact length cannot be measured.
(61) Specimen 4704 (SMF Be 1861) is preserved in Baltic amber. It is accessible in a dorsal view (Figure 49C,D). No clear camouflaging cloak is apparent. The antenna bears no prominent seta distally. Each tarsus of the trunk appendages carries a trumpet-shaped empodium. No prominent protrusions on the back are apparent, but there are setae along the trunk. The overall length of the larva is 1.31 mm.
(62) Specimen 4753 (CCHH 1786-3) is preserved in Baltic amber. It is accessible in dorsal (Figure 50A,B) and ventral view (Figure 50C). No camouflaging cloak is apparent. The head is well accessible in dorsal view (Figure 50D). The antenna bears no prominent seta distally. Each tarsus of the trunk appendages carries a trumpet-shaped empodium (Figure 50E). No prominent protrusions on the back are apparent. The overall length of the larva is 5.45 mm.

### 3.2. Shape Analysis

The analysis of the head shape resulted in four effective principal components (File S3). They together explain 95.37% of the overall variation (for the full results of the shape analysis, see Files S3–S5).

PC1 explains 79.69% of the overall variation. It is strongly influenced by the relative length of the stylets. A low value indicates shorter stylets, a high value indicates longer stylets (File S4).

PC2 explains 9.24% of the overall variation. It is strongly influenced by the posterior rim of the head capsule. A low value indicates a concave posterior rim, a high value indicates a convex posterior rim (File S4).

PC3 explains 3.90% of the overall variation. It is strongly influenced by the anterior rim of the head capsule. A low value indicates a convex anterior rim, a high value indicates a concave posterior rim (File S4).

PC4 explains 2.55% of the overall variation. It is strongly influenced by the proximal region of the stylets at the transition to the head capsule. A low value indicates a concave shape, a high value indicates a convex shape (File S4).

### 3.3. Size-Shape Correlation

As PC1 was so strongly dominating (almost 80%), we looked for a possible correlation of it to size. We did this for different types of larvae (explained in more detail in the discussion section). For type 1 larvae (for differentiation of larval types, see below), such a correlation is weak with an R^2^-value of 0.40. For type 2 larvae, only two specimens were available, providing no coefficient of determination. For type 3 larvae, the correlation is quite strong with an R^2^-value of 0.88. For type 4 larvae, the correlation is weaker with an R^2^-value of 0.63. Finally, for Hemerobiidae-type larvae there is a strong correlation with an R^2^-value of 0.95.

## 4. Discussion

### 4.1. What Is an Aphidlion?

As outlined, aphidlions are larvae of Chrysopidae and Hemerobiidae, and these two groups seem not to be closely related to each other. What does that mean for the term aphidlion? What does it in fact refer to?

We already know that, from the ecological functional side, the term refers to a larva with specific feeding habits. Yet, apparently it also refers to a certain combination of morphological characters, otherwise we could not recognise a fossil as “aphidlion-like”. These characters include a simple head with curved, rather slender, but toothless stylets, well-developed antennae and labial palps, and a simple spindle-shaped body.

Most of these characters very likely characterise a rather basal node within Neuroptera, which seems so far unnamed, and is namely the group of lacewings excluding Coniopterygidae, Nevrorthidae, Sisyridae, and Osmylidae (following the phylogeny of [34]). When assuming such a morphology for the larva of this node, this would then demand for the evolution of novelties in several lineages.

Simple-curved toothless stylets would become derived (“lost”) in the lineages of Dilaridae, Mantispidae + Berothidae, and Myrmeleontiformia. A spindle-shaped body is retained in most lineages; within Myrmeleontiformia, a broader shape has evolved (see discussion in [16,24]). This scenario seems in fact more likely than assuming any other morphology for the mentioned node within Neuroptera.

“Aphidlion” is therefore likely a kind of ecological category, but it also refers to a rather unspecialised terrestrial predator that likely represents the ancestral condition of larval morphology and ecology for a large group within Neuroptera. Within the group, the larvae evolved more specialised morphologies and, coupled to this, strategies, such as pit traps (within Myrmeleontidae; e.g., [61,62] and references therein), burrowing (as in Ithonidae, Nemopterinae, or Dilaridae, e.g., [63,64,65]), or types of parasitism (within Mantispidae; e.g., [66,67,68,69,70]). The fact that an aphidlion-like morphology is therefore likely characterised by a number of plesiomophies makes a phylogenetic interpretation of the specimens discussed here partly more challenging.

### 4.2. Identity of the Fossils

For identifying the closer relationships of very well-preserved fossil lacewing larvae, phylogenetic approaches were quite successful [14,15]. Yet, with less well and less completely preserved specimens, such approaches are much more challenging. Given that many of the specimens analysed here are indeed much less complete, and in some cases very distorted, we consider it as more realistic to use a simpler comparative approach, as for a phylogenetic analysis too many character states would have to be scored with “?”. This approach is common [7,8,9,10,12,13] and reasonably sufficient for the further reaching analysis [20,21,22,23,24,25].

Many of the Cretaceous larvae discussed here have already been interpreted as at least closely related to Chrysopidae [7,8,9,10,11,12,13] and many of the new fossils resemble these in critical aspects. Moreover, some of the younger fossils have definitely been identified as representatives of Chrysopidae and Hemerobiidae [49,52,71]. All the new fossils reported here clearly have an overall aphidlion-like appearance. For further reaching discussion, we will compile several similar appearing Cretaceous specimens to morphotypes and compare characters of these in more detail.

### 4.3. Differentiation of the New Cretaceous Material into Coarse Morphotypes

Many of the Cretaceous fossils bear protrusions on the dorsal side, similar to many fossil larvae that have been generally interpreted as relatives of Chrysopidae [7,8,9,10,11]. They may either be ingroup representatives of Chrysopidae or derivatives of the lineage towards the group (“stem-lineage”). Some of these are not very well preserved and do not provide further clues in order to group them into more distinct morphotypes; the better preserved ones allow one to differentiate three different types of larvae with such protrusions:

(1) Larvae of type 1 have relatively long protrusions on the anterior and posterior trunk, which are all about the same length. Many of the not-that-well-preserved specimens may represent type 1 larvae, yet they cannot be reliably interpreted.
(2) Larvae of type 2 have relatively long protrusions on the anterior trunk, but even longer ones on the posterior trunk.
(3) Larvae of type 3 have relatively long protrusions on the anterior trunk, but rather short ones on the posterior trunk.

Another type of larvae, type 4, lacks dorsal protrusions. These larvae have shorter lateral protrusions and relatively long stylets. Their trunk region is often much better preserved than those of the first three morphotypes. None of these larvae seem to bear a camouflaging cloak.

The last distinct type has a slender body, no protrusions, and shorter stylets. This feature is reminiscent of the larvae of Hemerobiidae. Therefore, we will use “Hemerobiidae-type” as a reference. Details further supporting or contradicting this interpretation will be given in the following.

### 4.4. Identity of the Fossils: Stylet Shape and Head Shape

The stylets of all new specimens are gently curved and rather slender. In many ingroups of Neuroptera, the stylets are more or less straight (Osmylidae, Sisysridae, Coniopterygidae, Dilaridae, Berothidae, many larvae of Mantispidae), or curved, but shorter and stouter (other larvae of Mantispidae, Ithonidae, Nemopterinae). In some neuropteran ingroups, the stylets are proximally straight, but curved distally (Nevrorthidae, [72]; *Ankyloleon*, [15]). Simple-curved stylets, as in the here discussed fossils, occur in Chrysopidae, Hemerobiidae (although they are often shorter than those of Chrysopidae), some fossil larvae of Mantispidae, and many ingroups of the larger group Myrmeleontiformia. The ground pattern of Myrmeleontiformia appears to be characterised by stylets that carry teeth [14,16] unlike in the fossils discussed here, yet in some lineages such teeth seem to have been secondarily lost (Psychopsidae: [21]; some later stage larvae of Crocinae: [16,24]). Furthermore, it is likely that curved stylets are an ancestral state (see above).

The head capsule shape of most of the new fossils clearly differs from that of long-nosed antlions (larvae of Psychopsidae) and long-necked antlions (larvae of Crocinae). Long-nosed antlions have a prominent labrum region projecting forward from the head capsule [21], which is not the case for the fossils reported here. Long-necked antlions either possess a rather triangular/trapezoidal head or a roughly rectangular head with prominently drawn out postero-lateral corners (“temples”) [24], which is different in most of the fossils reported here. An ingroup position of these within Myrmeleontiformia seems therefore unlikely.

### 4.5. Identity of the Fossils: Other Head Appendages

Other prominent head structures are the antennae and the labial palps. The antenna of many fossils, especially of types 1–3, bears a prominent distal seta (e.g., Figure 5D, Figure 13C, Figure 25D, Figure 31A, Figure 33E and Figure 47A). Such a seta is known in larvae of Chrysopidae and Hemerobiidae, yet comparable setae have been recognised in other groups (Psychopsidae: [21]) and may therefore not be a strong indicator. Moreover, other aspects of the antenna seem not to provide a strong signal. Modern larvae of both Hemerobiidae and Chrysopidae have antennae with three elements, with the second being the longest [38]. In many specimens of all types, this seems to be the case as well (e.g., Figure 1F, Figure 2C, Figure 5D, Figure 7E, Figure 9A,L, Figure 13C, Figure 15A, Figure 23A, Figure 25D, Figure 29D, Figure 30D and Figure 33D). The long middle element appears to be flexible to a certain degree and possesses a kind of pseudo-annulation, at least in well preserved specimens of different types (Figure 41A and Figure 42A; also observed in younger fossil larvae of Chrysopidae, Figure 49). Finally, in one type 4 larva the antenna appears to have four elements (Figure 34A).

The labial palps of larvae of Chrysopidae consist of three elements, with the second one being the longest. The labium of larvae of Hemerobiidae often has a proximally conjoined part projecting forward, which seems rather unique within Neuroptera. Moreover, in Hemerobiidae, the palps have three elements, with the third one being the longest; in some modern larvae this element appears swollen (e.g., [73]; see also the discussion in [25]). Such a condition is found in some of the new fossils, indicating that these are larvae of the group Hemerobiidae, or at least closely related to this group (Figure 9H and Figure 12E). In one specimen interpreted as a Hemerobiidae-type, 4842 (PED 0441), elements 2 and 3 of the labial palp have about the same length, with element 2 appearing only slightly longer (Figure 21D). This condition could still indicate a closer relationship to Hemerobiidae. Another problematic specimen, 4832 (PED 0248), clearly shares characters with modern larvae of Hemerobiidae (Figure 11E–J); yet it also resembles fossil larvae that have been interpreted as immatures of mantis lacewings (Mantispidae; [25]). As pointed out in [25], there are certain similarities between larvae of Mantispidae and Hemerobiidae that can make a distinction of fossil representatives challenging. For the moment, we tentatively treat the specimen here as one of the Hemerobiidae-type, yet observe the necessity for a larger-framed comparison also including Mantispidae in the future.

### 4.6. Identity of the Fossils: Empodia and Claws

Some of the fossils bear prominent trumpet-shaped empodia as adhesive structures on their trunk appendages ([74]; e.g., type 1: Figure 2C, Figure 11D, Figure 19E and Figure 38D; type 2: Figure 1E; type 3: Figure 14D, Figure 44E and Figure 46E), some also claws, some neither nor, i.e., the tarsus itself appears claw-like, but does not bear separate small distal claws; this last combination seems to be restricted to the larvae of type 4 (Figure 7E, Figure 9D,M and Figure 23D). One specimen of type 4 bears paired claws (Figure 34E); it is the same one, 4859 (PED 0952), that also differs in antenna structure from the others (see above). Another specimen of type 4, 4870 (PED 1301), bears claws and empodia (Figure 41D). 

The empodium seems to have evolved within Neuroptera, but lost again within Myrmeleontiformia. Prominent empodia can, for example, be found in larvae of Chrysopidae, Hemerobiidae, Mantispidae, also Psychopsidae, and extinct representatives of Myrmeleontiformia, such as larvae of *Macleodiella* Badano and Engel, 2018 [14,20,75]. Within Myrmeleontiformia, a derived group seems to be characterised by the loss of this structure (e.g., discussion in [76]).

Yet, the case is in fact a bit more complicated. Extant larvae of Chrysopidae have empodia only in larval stages 2 and 3 [26,38], but not in larval stage 1, which only bears claws [40]. Larvae of Hemerobiidae bear empodia only in stage 1 larvae, but not in stage 2 and 3 [38,40]. Any absence of empodia in a fossil can therefore not exclude its possible relationship to Chrysopidae and Hemerobiidae, but might instead indicate a state of a specific larval stage.

### 4.7. Identity of the Fossils: Processes on the Back and Camouflaging Cloak

Some extant larvae of Chrysopidae have dorsal processes and use these to attach a camouflaging cloak [29]. Larvae of Hemerobiidae are not known to have such structures, nor to bear a camouflaging cloak [38,77,78]. Yet, processes on the back and camouflaging cloaks are also known in some other ingroups of Neuroptera [11,14,79].

The structure of the processes in the new fossils differs from those of other groups, but also from those in the modern-day larvae of Chrysopidae. Still, it seems most parsimonious to assume that all Cretaceous specimens discussed here with such processes are closely related to Chrysopidae, basically following earlier interpretations of comparable fossils (e.g., [9,10,11]).

As to be expected in such a larger sample, there is quite some variation within the material. It seems likely that the sample contains different larval stages (see further below for this aspect), but probably also different species. As the state of preservation in many of these larvae is at best challenging, we refrain from erecting formal species based on these specimens. We hope that expanding the data set further in the future will allow for a stricter phylogenetic approach, as performed in Badano et al. [14,15].

### 4.8. Identity Summary

Most specimens reported here seem to be relatives of modern green lacewings (Chrysopidae). Types 1–3 are characterised by the prominent processes of the back and hence are quite similar to the modern aphidlions of Chrysopidae. Moreover, type 4 specimens strongly resemble modern green lacewing aphidlions, but are clearly of a non-camouflaging type. The differences within these specimens (as outlined above) may indicate that this type in fact represents at least two different species. Indeed, the fossils of the Hemerobiidae-type possess characters that further support an interpretation of these larvae as at least closer relatives of these lineages.

### 4.9. Ontogenetic Effects

**Figure 51:**
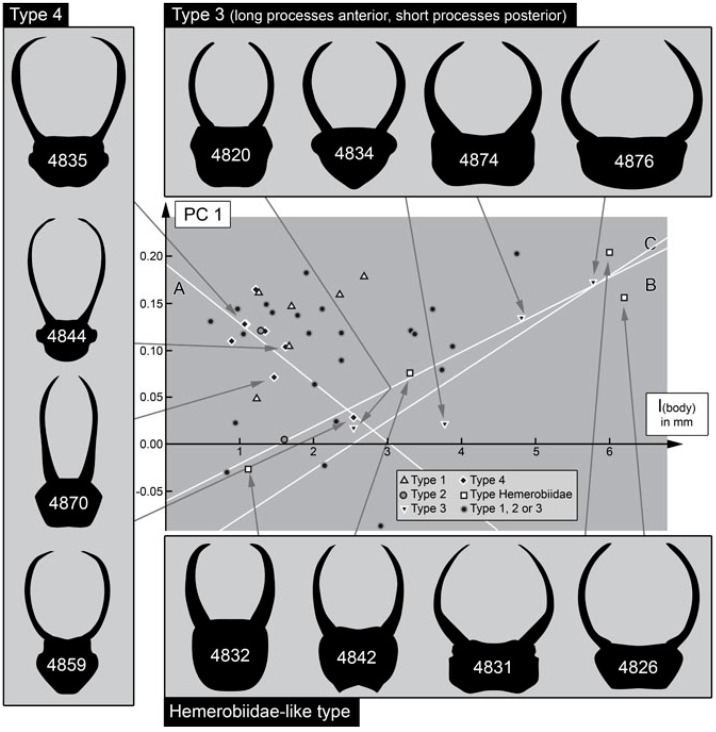
Scatter plot of PC1 vs. size. When distinguishing types, ontogenetic trajectories become apparent. Type 4 exhibits an ontogenetic change from broader heads to narrower ones. Type 3 and Hemerobiidae-like type exhibit an ontogenetic change from narrower heads to very broad heads.

In earlier quantitative studies of lacewing larvae, no real effects of shape and size could be recognised (e.g., [21], figure 27C; [22], figure 12). This is different in the data set here, at least when considering the different morphotypes separately (Figure 51). When plotting PC1 over size, type 3 larvae and Hemerobiidae-type larvae demonstrate a strong correlation of shape and size. In both cases, smaller specimens have rather square-shaped heads (in a dorsal view); larger specimens have more broad rectangular ones. This correlation indicates an ontogenetic change from a more square-shaped head outline to broad head shapes in these two types of larvae. In type 4 larvae, the trajectory is quite the opposite: smaller specimens have broader heads, larger specimens have progressively more square-shaped ones.

For extant aphidlion species, there seems to be no clear overall pattern. In some species, there seems to be no overall change (e.g., [80]), but also a change from a broader head to a less broad one occurs (e.g., [81]), and hence, is similar to type 4 fossil larvae. While the same pattern seems to also be present in other species [82,83,84], in those species the visible part of the head (see analysis) becomes in fact functionally broader over ontogeny as the head is more strongly retracted into the anterior trunk. It is therefore well possible that the observed patterns in the fossils relate to true ontogenetic sequences. It is still not possible to recognise distinct larval stages (instars), more specimens will be necessary to reliably identify these. There can be no clear signal for type 2 larvae, as only two specimens were included in the study. The merely weak correlation of shape and size for type 1 larvae might indicate that this type in fact includes several species.

### 4.10. Quantitative Comparison: Coarser Frame

**Figure 52:**
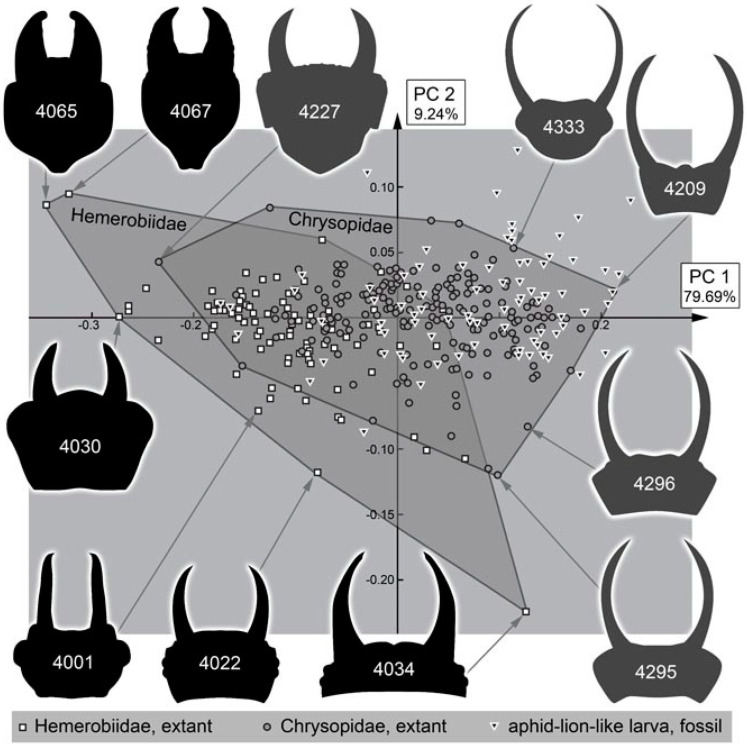
Scatter plot of PC2 vs. PC1 of head shapes of extant and fossil aphidlions and aphidlion-like larvae. Polygons mark areas occupied by extant representatives of Chrysopidae (head shapes exemplified by grey outlines) and Hemerobiidae (head shapes exemplified by black outlines).

Comparing the head shapes of the different groups included here reveals that all have large overlapping areas when plotting them in two dimensions. No group separates strongly from the others, yet certain differences can be well recognised (Figure 52). Extant green lacewing aphidlions plot more in the middle of the morphospace; extant brown lacewing aphidlions largely overlap with green lacewing aphidlions, but quite a few specimens also plot outside the range of the latter, more to the left and the bottom of the morphospace.

The fossil larvae plot largely within the range of green lacewing aphidlions (Figure 52). Only a single one plots outside this area and within the area occupied by brown lacewing aphidlions. This fossil is from the Eocene and was identified as a clear representative of Hemerobiidae. In general, all fossils from the Miocene and Eocene plot within the range of the modern larvae. Moreover, many of the larvae from the Cretaceous plot within the area occupied by modern larvae, more precisely the area occupied by green lacewing aphidlions. Yet, quite a few of them also plot outside the area occupied by modern larvae, mostly above and slightly to the right, indicating wider heads than in any modern aphidlion.

### 4.11. Quantitative Comparison: Morphospace of Changes of Green Lacewings through Time

**Figure 53:**
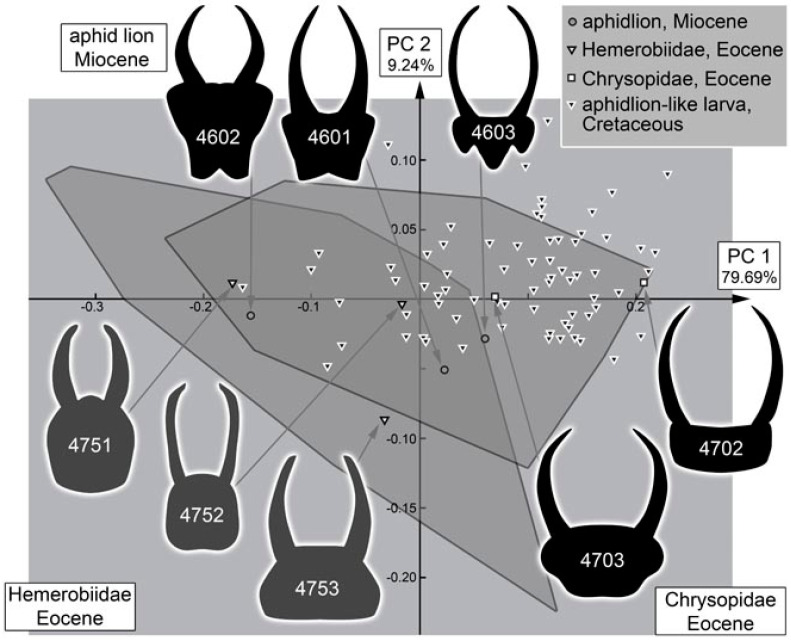
Scatter plot of PC2 vs. PC1 of head shapes of extant and fossil aphidlions and aphidlion-like larvae, continued. Polygons mark areas occupied by modern representatives of Chrysopidae and Hemerobiidae (as in Figure 52), with data points of extant specimens omitted. Head shapes of Hemerobiidae from the Eocene are exemplified by grey outlines and head shapes of Chrysopidae from the Eocene and of (presumable) Chrysopidae from the Miocene are exemplified by black outlines. Note that all larvae from the Eocene and Miocene plot within the area of the modern forms.

The comparison of the different time slices is partly complicated by the fact that the sample sizes for the Miocene and Eocene are much smaller than those of the Cretaceous and especially the modern fauna (Figure 53). The difference is so expressed that even a sample size correction (as e.g., in [21] for a similar analysis) cannot easily be applied. It may not be surprising that there are so few finds in the Miocene, as there are in general few finds of lacewing larvae in the Miocene ([49]). However, the low number of preserved larvae in the Eocene appears unusual. Currently, we have no explanation for this low number, but we cannot exclude that it reflects a truly lower abundance in the fauna of this geological period.

**Figure 54:**
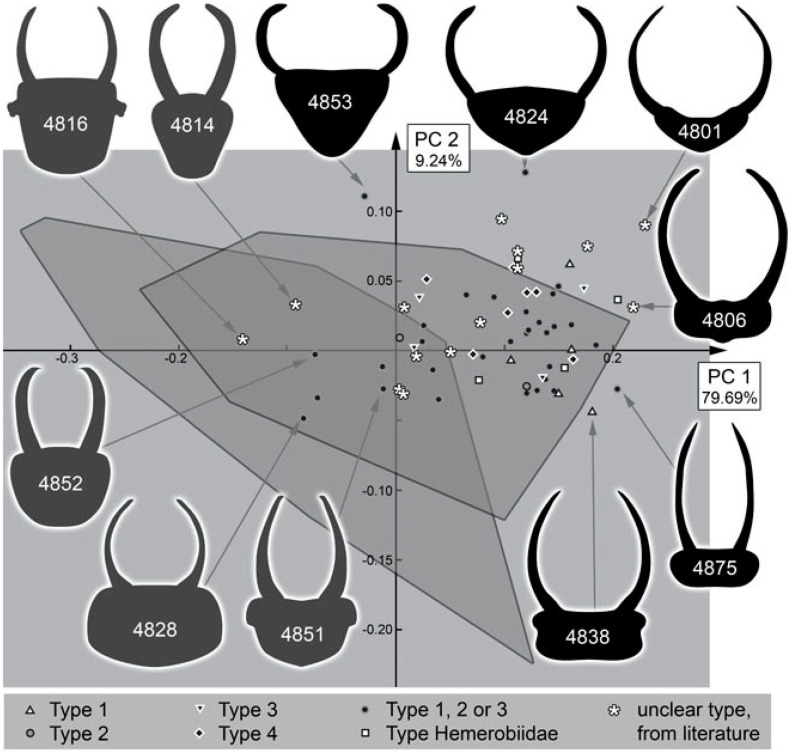
Scatter plot of PC2 vs. PC1 of head shapes of extant and fossil aphidlions and aphidlion-like larvae, continued. Polygons mark areas occupied by modern representatives of Chrysopidae and Hemerobiidae (as in Figure 52); data points of extant, Miocene and Eocene specimens are omitted. Note that many larvae from the Cretaceous plot well within the area of the modern forms (head shapes exemplified by grey outlines), yet some do not (head shapes exemplified by black outlines).

We can therefore mostly recognise that the fossils from the Miocene and Eocene fall within the area of the morphospace occupied by modern larvae. It is, however, noteworthy that the range of the green lacewing aphidlions in the Miocence and Eocene spans more or less the entire space occupied by modern green lacewing aphidlions in the PC1. This large range indicates that although the overall number of specimens is rather low, the overall morphological diversity of green lacewing aphidlions in the Eocene/Miocene was already about as large as today. Moreover, for the Cretaceous, the area occupied by modern green lacewing aphidlions was already largely occupied, at least along the PC1 (Figure 54).

**Figure 55:**
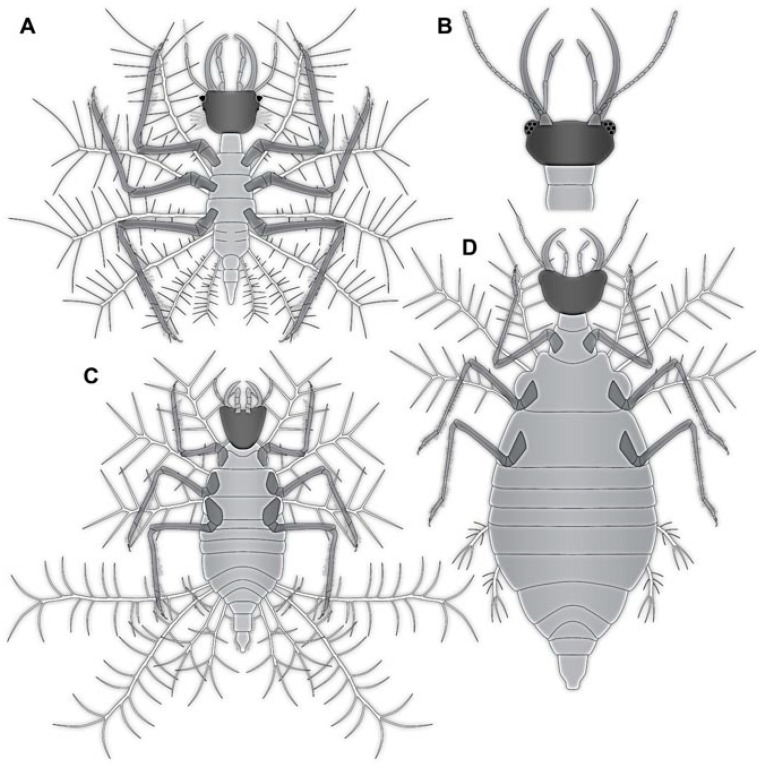
Restorations of some aphidlions, representing major types with processes. (**A**) Type 1, based on BUB 3066 (Figure 2). (**B**) Unclear type, based on BUB 3347 (Figure 3); note the strongly rectangular head. (**C**) Type 2, based on BUB 3060 (Figure 1). (**D**) Type 3, based on BUB 3361 (Figure 6).

The larvae in the Eocene and Miocene already have a very modern appearance and are indeed likely representatives of modern lineages. The Cretaceous larvae are not only similar in head shapes, but also partly in possessing structures on the back to carry a camouflaging cloak. Yet, in the details these Cretaceous larvae are quite different from the modern ones. For example, the head is much more distinctly set off in the fossils; moreover, the exact morphology of the camouflage-cloak-carrying processes is quite different (Figure 55).

These morphologies indicate that the fossils performed a very similar ecological role, yet are not closely related to the modern forms. We therefore assume that the Cretaceous species, probably entire lineages, went extinct, possibly at the end of the Cretaceous, and became ecologically replaced by the modern green lacewings before the Eocene. This combination of a severe extinction of some lineages in combination with a diversification of another lineage, basically substituting the extinct forms, is generally addressed as “faunal turnover”. Yet, this expression is usually used to address taxonomic changes of a community.

### 4.12. Quantitative Comparison: Morphospace of Changes of Brown Lacewings through Time

For brown lacewing aphidlions (Hemerobiidae), the case is different. The area exclusively occupied by modern brown lacewing aphidlions (not overlapping with green lacewing aphidlions) is basically unoccupied by fossils, besides a single specimen from the Eocene (Figure 53). The basic absence of these shapes in the fossil record as well as the rather low abundance indicates that the morphological diversification of brown lacewing aphidlions (with rather short and stouter stylets) is a more modern event. At least it seems that there is an increase in the morphological diversity of larvae in the lineage of Hemerobiidae since the Cretaceous.

It is unlikely that this observation is due to a preservation bias. Brown lacewing larvae nowadays have a quite similar lifestyle to green lacewing larvae in the aspects of living on plants and hunting their preferred prey there. With this, they should not have had a lower preservation potential. One could speculate that larvae with such a morphology back in the Cretaceous had a different lifestyle that is making a preservation in amber less likely (see discussion in [24]). This would still mean that the modern brown lacewing aphidlions evolved the modern habits only later, again indicating a more recent diversification of these types of larvae. Alternatively, brown lacewing aphidlions may have been less abundant or restricted to certain geographic regions in the Mesozoic. Moreover, the adults are rather rare in Cretaceous ambers, including Myanmar amber, despite being intensely studied [85], and also, in modern ecosystems brown lacewing aphidlions seem to be rarer than green lacewing aphidlions.

### 4.13. Lacewing Diversity through Time: The Larval Side

It is well established that lacewings were more diverse in the past [1,2], which can also be recognised in the quantitative morphology of the larvae. Clear losses have been recognised in Psychopsidae [21] and Nymphidae [22], and significant changes in Crocinae [24]; in some groups, there seems to be no significant change (Berothidae: [25]), or the data were indecisive (Nevrorthidae: [72]; Nemopterinae: [23]; Dilaridae and Osmylidae: [25]). Only in one group investigated so far for this aspect, Mantispidae, was an increase in larval diversity recognised [25]. The group Chrysopidae has here no significant change over time (although a slight loss of extreme forms can be observed; Figure 54). Hemerobiidae is another one of the few examples where there is a recognisable increase in the diversity of larval forms over the last 100 million years. These findings demonstrate that an overall decline of diversity of Neuroptera is complex on the detailed level, with more significant losses in certain ingroups, more or less stable ingroups, and even diversifying in very few ones.

### 4.14. Camouflaging Lacewing Larvae

As apparent in the here presented data set, the most common types of aphidlion larvae in the Cretaceous are those with protrusions on the back (types 1–3) that allow them to carry a camouflaging cloak (Figure 55). It is still interesting to note that many of these larvae do not carry traces of such a cloak. Of the three larvae of Chrysopidae from the Eocene, all carry at least traces of their camouflaging cloak.

**Figure 56:**
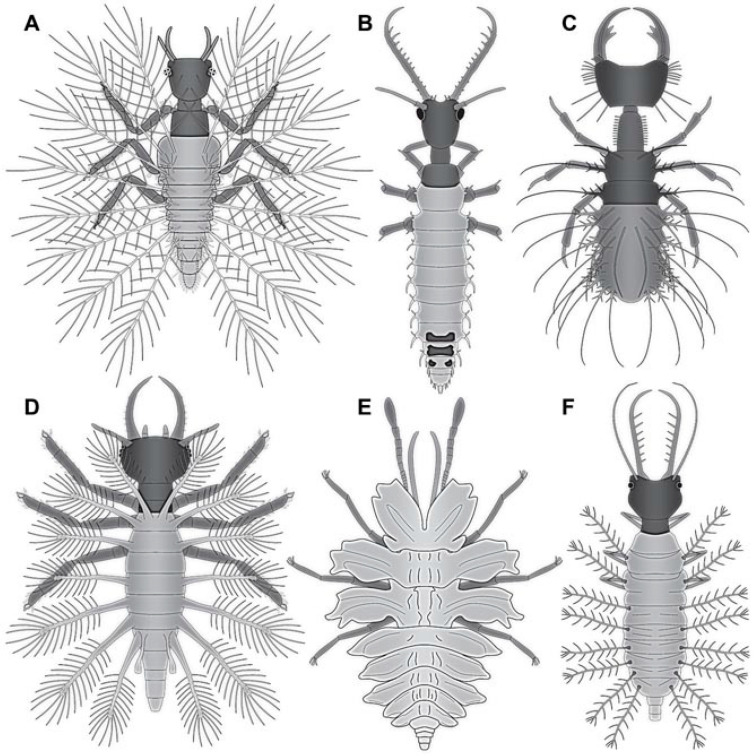
Different lacewing larvae with processes or other structures used for disguising themselves, not to scale. (**A**) *Tyruschrysa melqart* based on Pérez-de la Fuente et al. [9] with long processes similar to type 1. (**B**) Superfang larva from Haug et al. [18] with short processes similar to type 4. (**C**) *Electrocaptivus xui* based on Badano et al. [14] with proximally branched processes. (**D**) *Tragichrysa ovoruptora* based on Pérez-de la Fuente et al. [10] with long processes similar to type 1. (**E**) *Phyllochrysa huangi* based on Liu et al. [13] imitating a liverwort. (**F**) *Cladofer huangi* based on Badano et al. [14] with short-branched processes.

Larvae of type 4 have only short protrusions on the abdomen segments. These may still have acted as a support for a camouflaging cloak, as observed in other lacewing larvae (cf. [11]). Hence, the overwhelming majority of the aphidlion larvae in the Cretaceous seems to have carried, or at least was in principle able to carry, a camouflaging cloak. As indicated in earlier studies, many other lacewing larvae also seem to have used camouflaging strategies of one or the other types ([7,8,9,10,11,12,14,18,22,86]; Figure 56). Furthermore, in other lineages camouflaging behaviour has also been recognised, for bugs [11] or bark lice [87]. While in the latter case it is clear that the camouflage was used for a defensive purpose (to avoid predators), in the case of lacewings, larvae, and bugs, it might have been used defensively [86], aggressively (to get closer to possible prey), or both ways. In any case, the widespread presence of such strategies already indicates a quite complex interaction between the organisms 100 million years ago.

## 5. Conclusions

In summary, we observe in the case of Chrysopidae and their closer relatives almost no change in head shape over the last 100 million years. Nevertheless, in detail we observe:

(1) A certain loss of very extreme forms, most likely coupled to a loss of specific life habits (spider-association, [12]; mimicry, [13]);
(2) A loss of many further forms, especially of camouflaging larvae; yet this loss is factually compensated by:
(3) A diversification of the modern lineages of green lacewings and their larval forms.

In Hemerobiidae, the case is less clear due to a significantly smaller sample size. Still, the results indicate an increase in larval brown lacewings after the Cretaceous.

## Data Availability

All data from this study are available in this paper and the associated papers.

## Supplementary Materials

The following supporting information can be downloaded at: https://zenodo.org/record/6404287, File S1. Information on the specimens included in the analyses. Abbreviations: Eoc = Eocene; K = Cretaceous; Mio = Miocene; PC = principal component. File S2. Additional references for table in File S1 not referred to in main text. File S3. Results of the principal component analysis. File S4. Graphical representation of the factor loadings of the principal component analysis. File S5. Files resulting from the shape analysis, including chain codes, aligned shapes, and principal component analysis. Figure 1, Figure 2, Figure 3, Figure 4, Figure 5, Figure 6, Figure 7, Figure 8, Figure 9, Figure 10, Figure 11, Figure 12, Figure 13, Figure 14, Figure 15, Figure 16, Figure 17, Figure 18, Figure 19, Figure 20, Figure 21, Figure 22, Figure 23, Figure 24, Figure 25, Figure 26, Figure 27, Figure 28, Figure 29, Figure 30, Figure 31, Figure 32, Figure 33, Figure 34, Figure 35, Figure 36, Figure 37, Figure 38, Figure 39, Figure 40, Figure 41, Figure 42, Figure 43, Figure 44, Figure 45, Figure 46, Figure 47, Figure 48, Figure 49, Figure 50, Figure 51, Figure 52, Figure 53, Figure 54, Figure 55 and Figure 56 of high resolution are available in the supplementary materials.
